# A repertoire of visible light–sensitive opsins in the deep-sea hydrothermal vent shrimp *Rimicaris hybisae*

**DOI:** 10.1016/j.jbc.2025.110291

**Published:** 2025-05-26

**Authors:** Yuya Nagata, Norio Miyamoto, Keita Sato, Yosuke Nishimura, Yuki Tanioka, Yuji Yamanaka, Susumu Yoshizawa, Kuto Takahashi, Kohei Obayashi, Hisao Tsukamoto, Ken Takai, Hideyo Ohuchi, Takahiro Yamashita, Yuki Sudo, Keiichi Kojima

**Affiliations:** 1 Graduate School of Medicine, Dentistry and Pharmaceutical Sciences, Okayama University, Okayama, Japan; 2 Institute for Extra-Cutting-Edge Science and Technology Avant-Garde Research (X-Star), Japan Agency for Marine-Earth Science and Technology (JAMSTEC), Kanagawa, Japan; 3 Faculty of Medicine, Dentistry and Pharmaceutical Sciences, Okayama University, Okayama, Japan; 4 Research Center for Bioscience and Nanoscience (CeBN), Research Institute for Marine Resources Utilization, Japan Agency for Marine-Earth Science and Technology (JAMSTEC), Kanagawa, Japan; 5 School of Pharmaceutical Sciences, Okayama University, Okayama, Japan; 6 Atmosphere and Ocean Research Institute, The University of Tokyo, Chiba, Japan; 7 Department of Biology, Graduate School of Science, Kobe University, Kobe, Japan; 8 Department of Biophysics, Graduate School of Science, Kyoto University, Kyoto, Japan

**Keywords:** rhodopsin, opsin, G protein–coupled receptor, signal transduction, photoreceptor, vision, photobiology, vent shrimp, deep sea, molecular evolution

## Abstract

Unlike terrestrial environments, where humans reside, there is no sunlight in the deep sea. Instead, dim visible light from black-body radiation and bioluminescence illuminates hydrothermal vent areas in the deep sea. A deep-sea hydrothermal vent shrimp, *Rimicaris hybisae*, is thought to detect this dim light using its enlarged dorsal eye; however, the molecular basis of its photoreception remains unexplored. Here, we characterized the molecular properties of opsins, universal photoreceptive proteins in animals, found in *R*. *hybisae*. Transcriptomic analysis identified six opsins: three Gq-coupled opsins, one Opn3, one Opn5, and one peropsin. Functional analysis revealed that five of these opsins exhibited light-dependent G protein activity, whereas peropsin exhibited the ability to convert all-*trans*-retinal to 11-*cis*-retinal like photoisomerases. Notably, all the *R*. *hybisae* opsins, including Opn5, convergently show visible light sensitivity (around 457–517 nm), whereas most opsins categorized as Opn5 have been demonstrated to be UV sensitive. Mutational analysis revealed that the unique visible light sensitivity of *R*. *hybisae* Opn5 is achieved through the stabilization of a protonated Schiff base by a counterion residue at position 83 (Asp83), which differs from the position identified in other opsins. These findings suggest that the vent shrimp *R*. *hybisae* has adapted its photoreceptive devices to dim deep-sea hydrothermal light by selectively maintaining a repertoire of visible light–sensitive opsins, including the uniquely tuned Opn5.

Sunlight contains a wide range of light that reaches the surface of earth, including infrared, near-infrared, visible, and UV. Animals detect sunlight, especially in the visible and UV regions, using opsins, which are universal photoreceptive proteins responsible for visual and nonvisual physiological functions (*e*.*g*., regulation of circadian rhythms, seasonal sensing, and body color changes) (1, 2, 3). Opsins consist of a seven-transmembrane α-helical apoprotein and retinal as a chromophore. The retinal is covalently bound to a conserved Lys residue (Lys296 in the bovine rhodopsin [bovine Rh] numbering system) located in the seventh helix (helix VII) of the apoprotein through a Schiff base linkage. While retinal alone absorbs only UV light, opsins can absorb a wide range of light from UV to visible light (approximately 350–570 nm) in animals. In visible light–sensitive opsins, a protonated Schiff base is required for visible light sensitivity and is stabilized by a negatively charged residue(s) (*i*.*e*., Glu or Asp) called a counterion (4). Recent advances in cloning and sequencing technologies have enabled researchers to identify a variety of opsins and revealed that opsins are phylogenetically divided into several subfamilies that correlate well with functional classifications (*e*.*g*., G protein selectivity, photoreaction, spectral sensitivity, and retinal binding ability) (5, 6). In most subfamilies, opsins act as G protein–coupled receptors (GPCRs) containing 11-*cis*-retinal in the dark state (1, 2). Light absorption triggers photoisomerization from 11-*cis*- to all-*trans*-retinal, which leads to the formation of the G protein–activating state. The active states can couple to cognate heterotrimeric G proteins, such as Gi-, Gq-, and Gs-type G proteins, to trigger intracellular signal transduction cascades (1, 2). In contrast, two subfamilies of opsins (*i*.*e*., peropsin and retinal G protein–coupled receptor [RGR]/retinochrome) preferentially bind to all-*trans*-retinal in the dark state (1, 7). Light absorption triggers photoisomerization from all-*trans*- to 11-*cis*-retinal. These opsins are thought to act as retinal photoisomerases, supplying the 11-*cis*-retinal to the GPCR-type opsins. Since opsins function as the primary converters of environmental light information into intracellular signals in animals, researchers have sought to identify and characterize opsins in order to understand the molecular basis of photoreception across a variety of animals.

In contrast to terrestrial environments, sunlight cannot reach the deep sea because of scattering and absorption by water. While some deep-sea animal species have degenerated eyes, others have modified eye morphologies and spectral sensitivities of opsins that efficiently absorb weak light, primarily from bioluminescence produced by marine bacteria and animals, which exists even at these depths (8, 9). After the initial discovery of high-temperature deep-sea hydrothermal vents over 4 decades ago, more such vents have been discovered in the world’s oceans (10). In addition to visible light from bioluminescence, infrared light and low levels of near-infrared and visible light are present around these vents because of black-body radiation from the extreme heat (11, 12). While black-body radiation consists of visible light with a relatively long wavelength (>500 nm at 300 °C) (11), the bioluminescence from marine bacteria generally consists of blue light (approximately 440–500 nm) (8). Thus, dim visible light primarily from black-body radiation and bioluminescence is indeed present in the vicinity of deep-sea hydrothermal vents. These environments host unique communities with diverse endemic fauna, including several crustacean species (10) which feed on chemosynthetic microorganisms near the vents but must avoid the lethal heat (13). Some crustacean species (*e*.*g*., *Rimicaris exoculata* and *Bythograea thermydron*) have degraded eyestalks and corneas but have developed an enlarged eye located underneath the dorsal carapace to detect dim light (13, 14, 15, 16). In the process of forming the dorsal eye (also called the dorsal organ), image-forming optics have been lost while the rhabdomeric photoreceptor has enlarged and contains a high concentration of photoreceptive opsin molecules. Based on this background, it is believed that hydrothermal vent crustaceans recognize their distance from the lethal area near the vents and the feeding area containing marine bacteria by using opsins expressed in their eyes to detect the dim light from the vents and bacteria. It is crucial to reveal the molecular basis of this photoreception to understand how crustaceans have adapted to the unique photic environment of hydrothermal vent areas. Rimicarid shrimps, which live near vent fluids and chimneys in numerous biogeographic provinces, including the Mid-Atlantic Ridge, Indian Ocean, and Mid-Cayman Spreading Center (MCSC), are key members of hydrothermal vent communities (10, 13, 14). In this study, we aimed to identify opsin genes and characterize their molecular properties in *Rimicaris hybisae* living in hydrothermal vent fields of the MCSC (Fig. 1*A*) (17). Our transcriptomic analysis identified six opsins, and functional analysis using recombinant proteins revealed that five of these opsins showed light-dependent G protein activity, whereas the remaining opsin showed the ability to convert all-*trans*-retinal into 11-*cis*-retinal like photoisomerase. Notably, all the *R*. *hybisae* opsins, including one categorized in the Opn5 subfamily, showed visible light sensitivities, whereas most opsins in the Opn5 subfamily were typically UV sensitive. Furthermore, we found that the unique visible light sensitivity of *R*. *hybisae* Opn5 is due to the stabilization of the protonated Schiff base by the counterion residue at Asp83. Based on these results, we discuss the adaptation strategies of the vent shrimp *R*. *hybisae* to the deep-sea environment.

**Figure 1:**
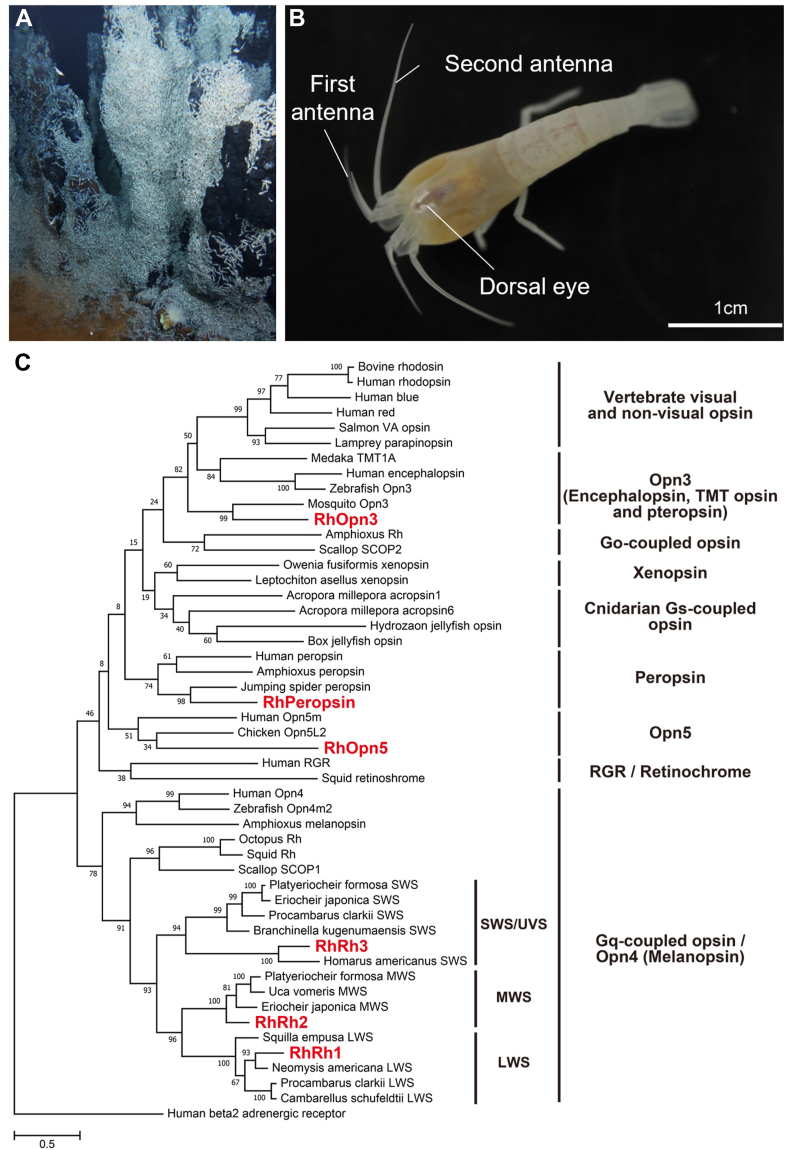
**Morphology of the dorsal eye of *Rimicaris hybisae* and phylogenetic tree of opsins**. *A*, aggregation of *R*. *hybisae* on a chimney at the hydrothermal vent field in the MCSC. *B*, a live specimen of *R*. *hybisae*, showing dorsal eye morphology. *C*, phylogenetic relationship of opsins. *R*. *hybisae* opsins are highlighted in bold red letters. MCSC, Mid-Cayman Spreading Center.

## Results

### Morphology of the dorsal eye and identification of opsins in *R*. *hybisae*

We first analyzed the morphology of the dorsal eye to investigate whether *R*. *hybisae* has adapted its dorsal eye to the hydrothermal vent environment (Figs. 1*B* and S1). Toluidine blue-stained plastic sections of the dorsal eye showed an enlarged rhabdomeral segment filled with microvillar membranes (Fig. S1*A*, *arrow*). We also used transmission electron microscopy to examine the ultrastructure of the rhabdomeric photoreceptor (Fig. S1, *B–C*). The rhabdomeric photoreceptor cells are composed of well-organized arrays of microvilli. The multilayered microvillar arrays in the rhabdomeric photoreceptor are suitable for containing a large number of opsin molecules. Although the detailed morphology of the eye and photoreceptors could not be fully revealed because of sample degradation, the observed morphological characteristics are consistent with those in the representative hydrothermal vent shrimp *Rimicaris exoculata* (13, 15). This suggests that *R*. *hybisae* has adapted its eye morphology to the dim light conditions around hydrothermal vents.

We next performed *R*. *hybisae* transcriptome screening and identified six opsin sequences containing Lys296 as a conserved chromophore-binding site (Figs. 1*C* and S2). The sequences featured the E/DRY and NPxxY motifs at positions 134 to 136 and 302 to 306, respectively, which are highly conserved in GPCRs, including opsins (1). To determine the subfamily of the six opsin sequences, we performed a phylogenetic analysis and identified three Gq-coupled opsins (RhRh1, RhRh2, and RhRh3), one Opn3 (RhOpn3), one Opn5 (RhOpn5), and one peropsin (RhPeropsin) (Fig. 1*C*). Gq-coupled opsins are categorized as visual opsins that are generally expressed in rhabdomeric photoreceptor cells in the eye and that trigger visual signal transduction cascades, whereas Opn3, Opn5, and peropsin are categorized as nonvisual opsins (1, 5, 6, 18). In crustaceans, Gq-coupled opsins are subdivided into three groups: long-wavelength sensitive (LWS), middle-wavelength sensitive (MWS), and short-wavelength (SWS/UVS) (18). The three Gq-coupled opsins in *R*. *hybisae*, namely RhRh1, RhRh2, and RhRh3, belong to the LWS, MWS, and SWS/UVS groups, respectively (Fig. 1*C*). Expression analysis of opsin genes showed that *RhRh1*, *RhRh3*, and *RhOpn3* were expressed in the dorsal eye in at least one sample each (Fig. S3). In contrast, *RhRh2* was expressed in the brain but not in the dorsal eye, despite its classification as a visual opsin. The expression levels of *RhOpn5* and *RhPeropsin* were under the criterion (≥2 transcripts per million).

### G protein activation of *R*. *hybisae* opsins

**Figure 2:**
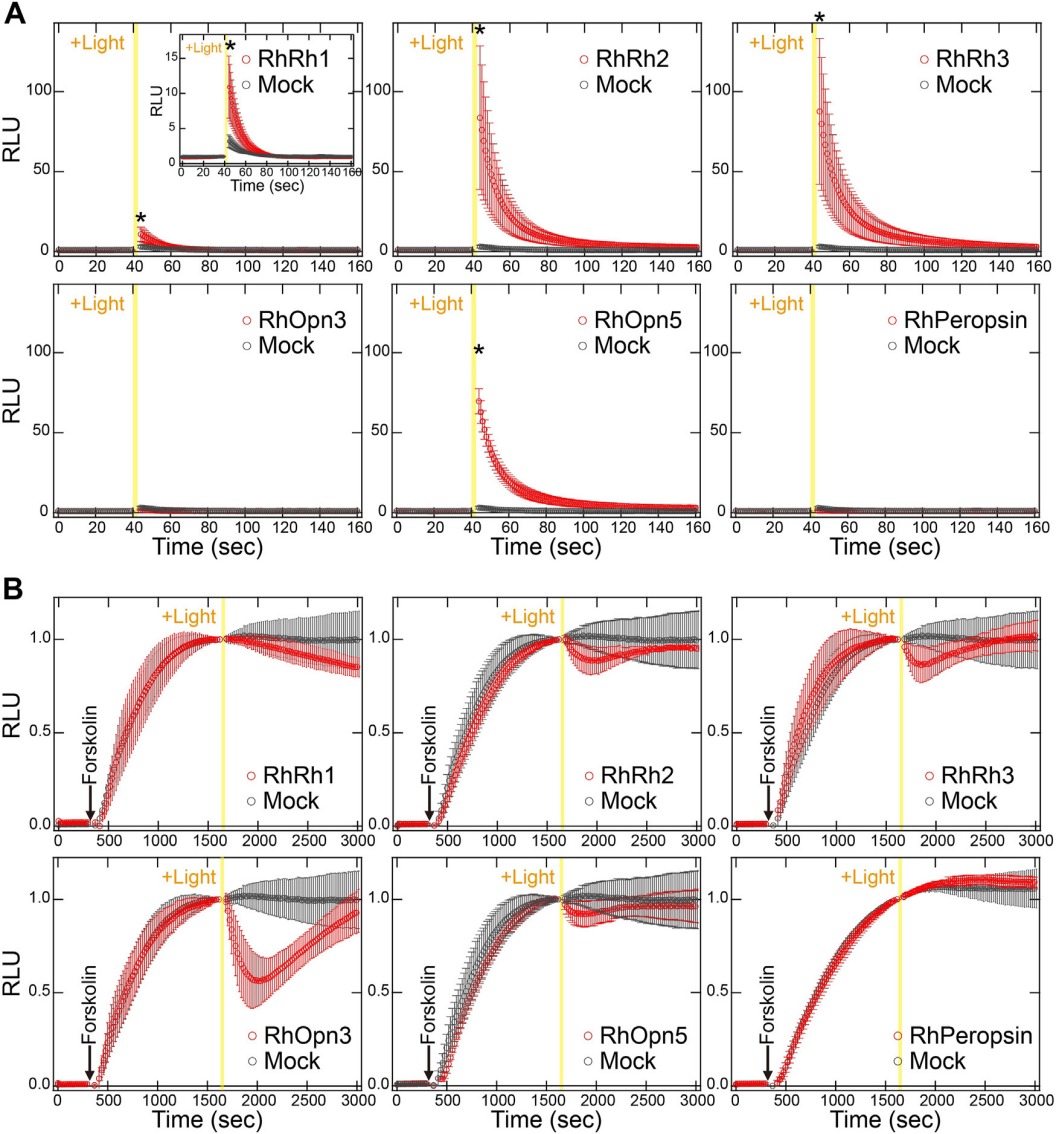
**Light-induced G protein activation by *Rimicaris hybisae* opsins**. *A*, light-induced changes in intracellular Ca^2+^ levels by *R*. *hybisae* opsins. The Ca^2+^ levels in RhRh1-, RhRh2-, RhRh3-, RhOpn3-, RhOpn5-, RhPeropsin-, and mock-transfected HEK293 cells were measured using the aequorin assay. Cells were irradiated by visible light covering wavelengths from 420 to 700 nm for 3 s. ∗ indicates a significant difference in luminescence values after irradiation between opsin- and mock-transfected HEK293 cells (*p* < 0.05; Dunnett’s test). *B*, light-induced changes in intracellular cAMP levels by *R*. *hybisae* opsins. The cAMP levels in RhRh1-, RhRh2-, RhRh3-, RhOpn3-, RhOpn5-, RhPeropsin-, and mock-transfected HEK293 cells were measured using the GloSensor cAMP assay. Cells were treated with 2 μM forskolin prior to irradiation with visible light covering wavelengths from 420 to 700 nm for 30 s. The *yellow vertical lines* in all panels of Figure 2 indicate the timing of visible light irradiation to the cells. Data are presented as the means ± SD from more than three independent experiments. HEK293, human embryonic kidney 293 cell line; RhOpn, *Rimicaris hybisae* Opn; RLU, relative light unit.

To investigate whether the six *R*. *hybisae* opsins function as photoactive proteins, we expressed the recombinant proteins in mammalian cultured cells and analyzed their light-dependent G protein–activation abilities. To improve the expression level of opsin proteins, we truncated 170 amino acid residues from the N terminus of the RhRh1 protein and 134 and 178 residues from the C termini of RhRh3 and RhOpn5, respectively. Opsins belonging to the Gq-coupled opsin, Opn3, and Opn5 subfamilies have been reported to activate Gq-, Gi-, and Gi-/Gq-type G proteins, respectively (2, 19, 20). In contrast, the protostome peropsin has been reported to show no significant G protein activity (21). Based on these reports, we investigated the ability to activate typical Gq-, Gi-, and Gs-type G proteins by detecting intracellular Ca^2+^ and cAMP levels using aequorin and GloSensor assays. First, we measured the intracellular Ca^2+^ level in mammalian cultured cells, as activation of the Gq-type G protein increases intracellular Ca^2+^ concentrations. We irradiated opsin-transfected cells with visible light covering wavelengths from 420 to 700 nm (Fig. S4). This wavelength range encompasses the visible light from black-body radiation (>500 nm) and bioluminescence (approximately 440–500 nm) (8, 11), which is present around hydrothermal vents in the deep sea. As a positive control, jumping spider rhodopsin-1 (JSR1), a Gq-coupled opsin, induced a significant increase in intracellular Ca^2+^ concentrations in a light-dependent manner (Fig. S5). Visible light irradiation significantly increased intracellular Ca^2+^ concentrations in RhRh1-, RhRh2-, RhRh3-, and RhOpn5-transfected cells, although to different levels (Fig. 2*A*). Since G protein βγ subunits generated from robust heterotrimer activation could result in an increase in intracellular Ca^2+^ concentrations as well as α subunit of Gq-type G protein, we analyzed the intracellular Ca^2+^ level in the presence of YM-254890 (a Gq inhibitor). The light-induced increases were depressed by adding YM-254890 (Fig. S5), which clearly indicates that these four opsins activate Gq-type G protein in a visible light–dependent manner. In contrast, no significant increase was observed in RhOpn3- or RhPeropsin-transfected cells. Second, we measured intracellular cAMP levels in mammalian cultured cells because the activation of Gi- and Gs-type G proteins decreases and increases intracellular cAMP concentrations, respectively. Bovine Rh, a Gi-activating opsin, decreased cAMP levels that had been increased by adding forskolin (an adenylyl cyclase activator), and this occurred in a light-dependent manner (Fig. S6*A*). Visible light irradiation caused marked, transient reduction in cAMP levels in RhOpn3-transfected cells (Fig. 2*B*). This transient reduction is likely because of the inactivation of the light-activated opsin (*e*.*g*., opsin bleaching and inactivation by arrestin). The light-dependent reduction in cAMP levels was inhibited by incubation with pertussis toxin (PTX), a Gi inhibitor (Fig. S6*A*). This indicates that RhOpn3 activates Gi-type G proteins in a visible light–dependent manner. The light-induced cAMP changes in bovine Rh (Fig. S6*A*) and RhOpn3 (Fig. 2*B*) were comparable. Since bovine Rh exhibits G protein activation signals comparable to or greater than those of ligand-binding GPCRs (22), we speculate that RhOpn3 likely exhibits comparable or higher Gi activation efficiency than Gi-coupled ligand-binding GPCRs. In addition, a small reduction in cAMP levels was observed in RhRh1-, RhRh2-, RhRh3-, and RhOpn5-transfected cells, suggesting that these opsins also activate Gi-type G proteins. Next, we measured cAMP levels with PTX to evaluate the activity of Gs activity. Jellyfish opsin (JelOp), a Gs-activating opsin, increased cAMP levels in a light-dependent manner (Fig. S6*B*). No significant increase in cAMP levels was observed in RhRh1-, RhRh2-, RhRh3-, RhOpn3-, RhOpn5-, or RhPeropsin-transfected cells (Fig. S6*B*), which implies that none of the *R*. *hybisae* opsins significantly activates Gs-type G proteins.

Furthermore, to confirm G protein heterotrimer activation using an alternative experimental system, we performed the NanoBiT-G protein dissociation assay (23). In this assay, G protein activation (Gα-βγ dissociation) is detected as a decrease in NanoLuc luminescence from the Lg-BiT fragment inserted with Gα and the Sm-BiT fragment fused with Gγ. We first assessed Gq activation using this system (Fig. S7*A*). As a positive control, JSR1 showed a light-induced decrease in luminescence, confirming its Gq activation (Gq dissociation). RhRh1-, RhRh2-, RhRh3-, and RhOpn5-transfected cells showed a transient, light-induced decrease in luminescence followed by a time-dependent increase, although the decrease observed in RhRh1-transfected cells was relatively small. These results indicate that these opsins activate Gq in a light-dependent manner, consistent with the aequorin assay results (Fig. 2*A*). On the other hand, RhOpn3-transfected cells also showed a light-induced decrease in luminescence, indicating Gq activation, although no such activation was detected in the aequorin assay (Fig. 2*A*). Next, we assessed Gi activation using the same system (Fig. S7*B*). As a positive control, bovine Rh exhibited a light-induced decrease in luminescence, indicating Gi activation (Gi dissociation). Similarly, RhRh2-, RhRh3-, and RhOpn3-transfected cells showed a marked light-induced decrease in luminescence. In contrast, a small decrease was observed in RhRh1- and RhOpn5-transfected cells. These results indicate that these opsins activate Gi in a light-dependent manner, with different activation efficiencies, which is consistent with the results of the GloSensor assay (Fig. 2*B*). In summary, with the exception of RhPeropsin, the opsins from *R*. *hybisae* exhibit G protein–activation ability (Gq and Gi for RhRh1, RhRh2, RhRh3, RhOpn3, and RhOpn5). Notably, visible light irradiation clearly induced their G protein activity, suggesting that these opsins function as visible light–sensitive opsins rather than UV-sensitive ones. As demonstrated by aequorin, GloSensor, and NanoBiT assays (Figs. 2 and S7), RhRh1 exhibited a weak signaling response, in contrast to the stronger responses observed for other *R*. *hybisae* opsins (*e*.*g*., RhRh2 and RhRh3). To investigate the cause of this weak response, we examined the expression levels of the opsins by Western blotting analysis (Fig. S8). RhRh1 (calculated molecular weight: 43 kDa) exhibited a faint band around 85 kDa, likely because of its oligomerization and the heterogeneity in post-translational modification within the cultured cells. In contrast, an intense band was observed around 20 kDa, which may result from denaturation. This suggests that RhRh1 is predominantly localized to intracellular membranes rather than the plasma membrane. We speculate that the reduced signaling response of RhRh1 is due to its limited localization to the plasma membrane. Therefore, to more accurately assess G protein activation efficiency of RhRh1, it will be necessary in future studies to enhance its localization to the plasma membrane, for example, by adding membrane trafficking signals to the *RhRh1* gene in the expression vector.

### Photoisomerase-like function of RhPeropsin

**Figure 3:**
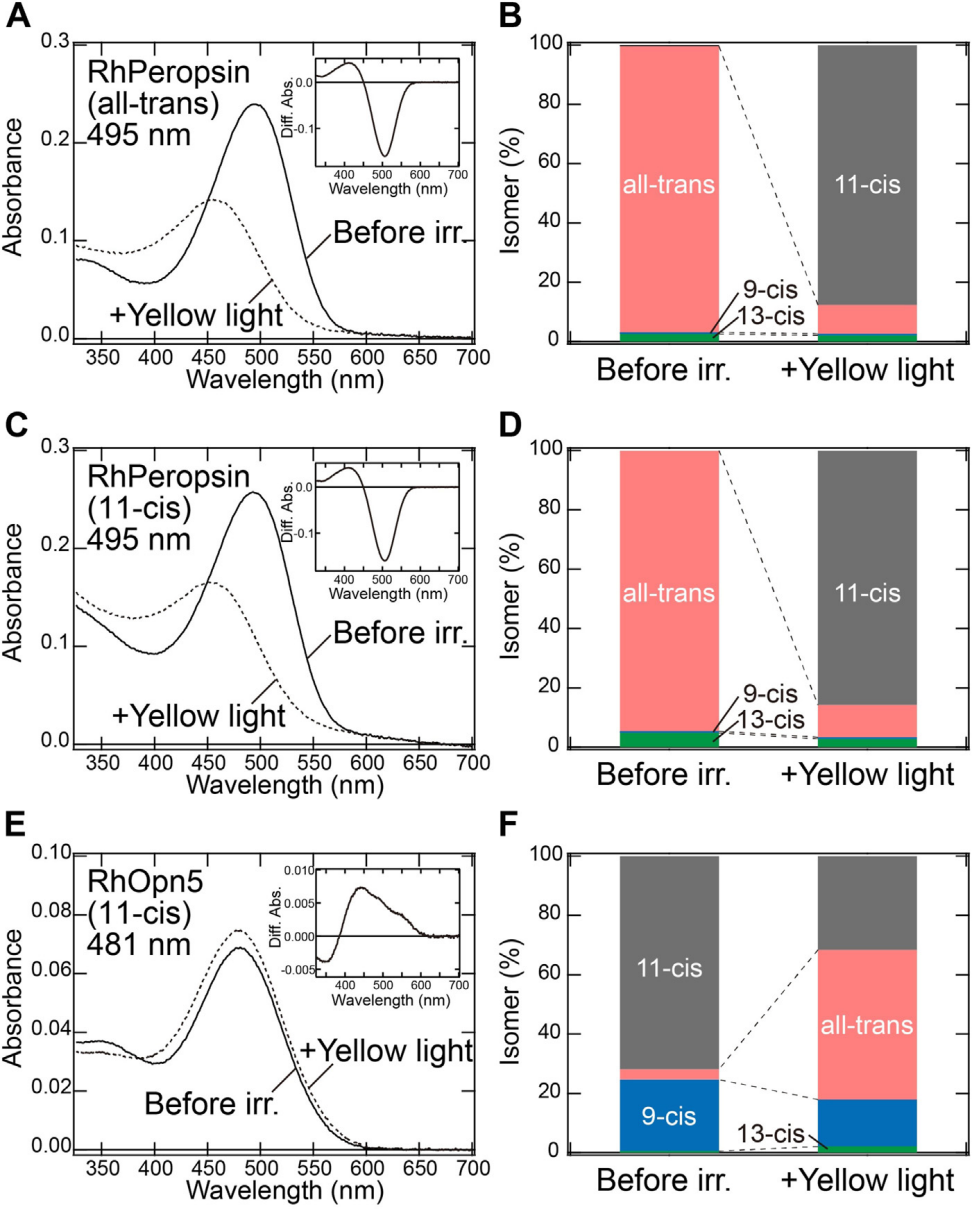
**Spectral sensitivities of RhPeropsin and RhOpn5**. *A* and *C*, absorption spectra of RhPeropsin reconstituted with all-*trans*- (*A*) and 11-*cis*-retinal (*C*). *Solid and dotted curves* indicate the spectra in the dark and after yellow light (>500 nm) irradiation, respectively. (*Inset*) The curves are the different spectra before and after yellow light irradiation. *B* and *D*, retinal configuration changes in RhPeropsin reconstituted with all-*trans*- (*B*) and 11*-cis*-retinal (*D*). Isomeric compositions of retinal in the dark and after yellow light irradiation were estimated by HPLC analysis (Fig. S9, *A*–*B*). *E*, absorption spectra of RhOpn5 reconstituted with 11*-cis*-retinal. *Solid and dotted curves* indicate the spectra in the dark and after yellow light (>480 nm) irradiation, respectively. (*Inset*) The curves are the different spectra before and after yellow light irradiation. *F*, retinal configuration changes in RhOpn5 reconstituted with 11-*cis*-retinal. Isomeric compositions of retinal in the dark and after yellow light irradiation were estimated by HPLC analysis (Fig. S9*C*). RhOpn5, *Rimicaris hybisae* Opn5.

To investigate whether RhPeropsin shows photoisomerase-like functions, we analyzed its photochemical properties. First, we purified the recombinant proteins of RhPeropsin reconstituted with all-*trans*-retinal. The absorption spectrum of purified RhPeropsin with all-*trans*-retinal showed an absorption maximum (λmax) at 495 nm in the visible light region (Fig. 3*A*). Analysis of the retinal configurations using HPLC revealed that the RhPeropsin proteins predominantly bound all-*trans*-retinal (Figs. S9*A* and S3*B*). Yellow light (>500 nm) irradiation induced a spectral blue shift, with a simultaneous increase in 11*-cis*-retinal and a decrease in all-*trans*-retinal in the pigments (Figs. 3, *A* and *B* and S9*A*). This implies that RhPeropsin converts all-*trans*-retinal to 11*-cis*-retinal upon visible light absorption. We also purified recombinant proteins of RhPeropsin reconstituted with 11-*cis*-retinal to investigate its binding affinity. The absorption spectrum of purified RhPeropsin showed a λmax at 495 nm, which is almost identical to that of the proteins reconstituted with all-*trans*-retinal (Fig. 3*C*). HPLC analysis confirmed that the RhPeropsin proteins predominantly bind all-*trans*-retinal rather than 11*-cis*-retinal (Figs. S9*B* and S3*D*). We speculate that RhPeropsin binds all-*trans*-retinal, which is generated by thermal isomerization of 11-*cis*-retinal in the culture medium when human embryonic kidney 293 (HEK293) cells expressing RhPeropsin are incubated with exogenous 11*-cis*-retinal. Yellow light (>500 nm) irradiation induced a similar spectral shift and conversion of all-*trans*-retinal to 11-*cis*-retinal as observed in the pigments reconstituted with all-*trans*-retinal (Figs. 3, *C* and *D* and S9*B*). These data clearly indicate that RhPeropsin exclusively binds all-*trans*-retinal in the dark and produces 11*-cis*-retinal from all-*trans*-retinal upon light absorption, similar to RGR/retinochrome and peropsin (24). Considering that RhPeropsin has a λmax at 495 nm in the visible light region, these results suggest that RhPeropsin functions as a visible light–sensitive photoisomerase to supply 11-*cis*-retinal in *R*. *hybisae*.

### Visible light sensitivities of *R*. *hybisae* opsins

Since only dim near-infrared and visible light is present around hydrothermal vents in the deep sea, we investigated whether the spectral sensitivities of *R*. *hybisae* opsins are tuned to these unique light conditions. It is noteworthy that most vertebrate Opn5 opsins (Opn5m and Opn5L2 subgroups), with the exception of the Opn5L1 subgroup, have been demonstrated to be UV sensitive (25, 26, 27), raising the crucial question of whether RhOpn5 also shows UV sensitivity despite the near absence of UV light in the deep sea. We successfully purified and obtained active recombinant proteins of RhOpn5, in addition to RhPeropsin, but failed to obtain photoactive forms of RhRh1, RhRh2, RhRh3, or RhOpn3. The absorption spectrum of RhOpn5 reconstituted with 11-*cis*-retinal showed a λmax at around 480 nm in the dark (Fig. 3*E*). HPLC analysis confirmed that this pigment predominantly bound 11-*cis*-retinal, with a small amount of 9-*cis*-retinal (Figs. S9*C* and 3*F*). Thus, RhOpn5 forms a blue light–sensitive pigment binding 11-*cis*-retinal in the resting state. Yellow light (>480 nm) irradiation induced a spectral change that formed a photoproduct with a similar λmax at around 480 nm, accompanied by the conversion from 11-*cis*- to all-*trans*-retinal (Fig. 3, *E* and *F* and S9*C*), which implies that the blue light–sensitive pigment is photoactive. Similar weak spectral shifts have also been reported in several invertebrate Gq-coupled opsins (*e*.*g*., squid rhodopsin and JSR1), which contain protonated all-*trans*-retinal (28, 29). In addition, deprotonated all-*trans*-retinal Schiff bases absorb in the UV region. These suggest that RhOpn5 contains protonated 11-*cis*-retinal in the dark and retains protonated all-*trans*-retinal after yellow light illumination. This spectroscopic analysis, together with the results of the G protein activity of RhOpn5 (Fig. 2), indicates that RhOpn5 functions as a visible light–sensitive GPCR and does not possess the UV sensitivity observed in most vertebrate Opn5 proteins.

**Figure 4:**
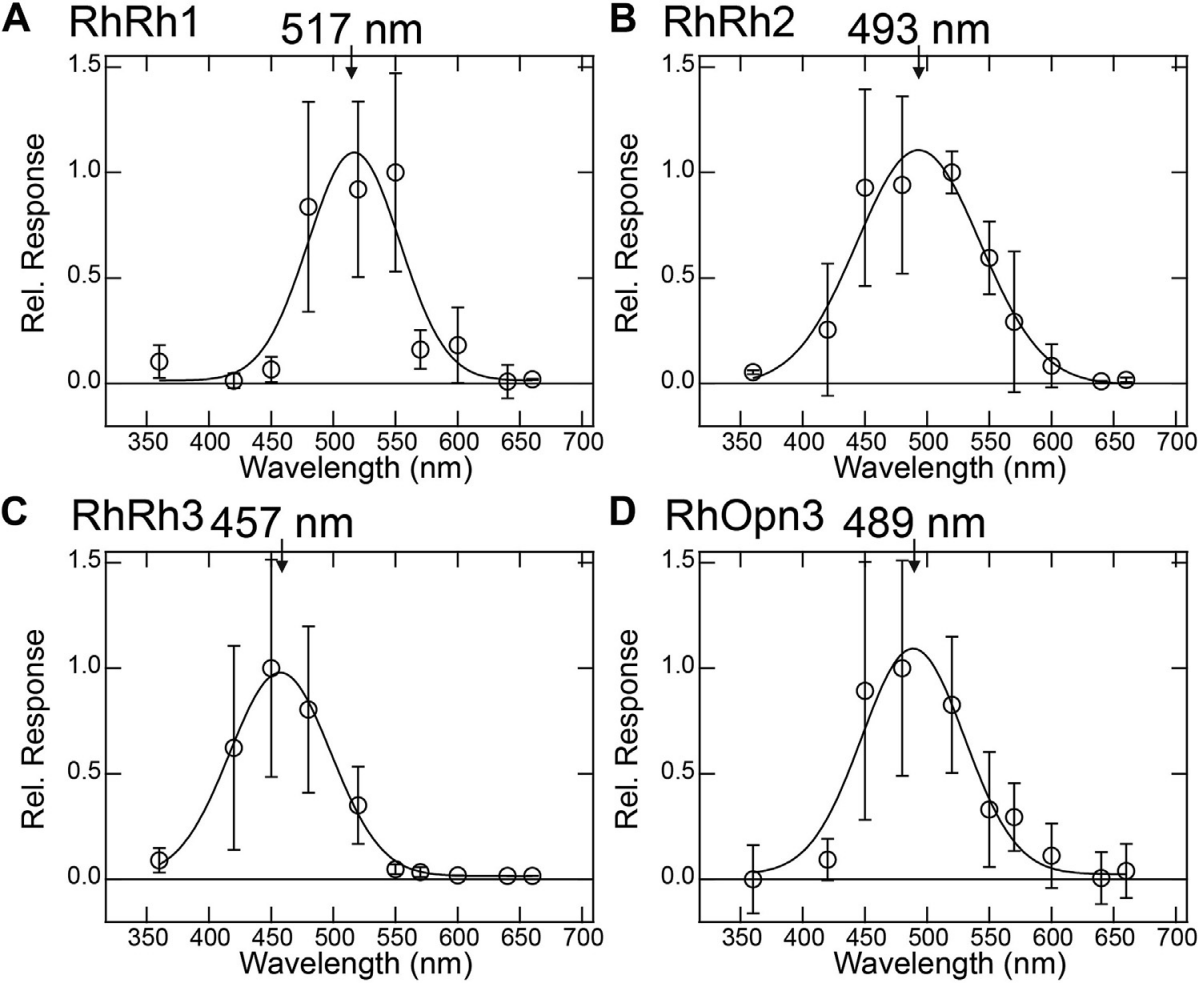
**Spectral sensitivities of RhRh1, RhRh2, RhRh3, and RhOpn3**. The relative light–induced responses of RhRh1 (*A*), RhRh2 (*B*), and RhRh3 (*C*) were determined by the amplitudes of light-induced changes in intracellular Ca^2+^ levels in RhRh1-, RhRh2-, and RhRh3-transfected HEK293 cells, respectively, and those of RhOpn3 (*D*) were determined by the initial slopes of light-induced changes in cAMP levels in RhOpn3-transfected HEK293 cells (see the *Experimental procedures* section for details). Data are presented as the means ± SD from more than three independent experiments. The responses were fitted with a Gaussian curve to estimate the peak wavelengths (517, 493, 457, and 489 nm for RhRh1, RhRh2, RhRh3, and RhOpn3, respectively). HEK293, human embryonic kidney 293 cell line; RhOpn3, *Rimicaris hybisae* Opn3.

Next, we evaluated the spectral sensitivities of other *R*. *hybisae* opsins based on the light-induced changes in intracellular Ca^2+^ levels (for RhRh1, RhRh2, and RhRh3) and cAMP levels (for RhOpn3) in mammalian cultured cells. To confirm the validity of this method, we analyzed the sensitivities of UV- and visible light–sensitive opsins, such as JSR1, chicken Opn5m (cOpn5m), bovine Rh (bovine Rh), and lamprey parapinopsin (LamPP). For JSR1 and cOpn5m, the increase in aequorin luminescence after irradiation with different colors of light was measured and plotted against the wavelength of light (Fig. S10, *A–B*). For bovine Rh and LamPP, the initial decreases in Glosensor luminescence slopes after irradiation with different colors of light were obtained and plotted against the wavelength of light (Fig. S10, *C–D*). By fitting the plots with a Gaussian function, we evaluated the peak wavelengths as 528, 360, 506, and 395 nm for JSR1, cOpn5m, bovine Rh, and LamPP, respectively. These values are roughly consistent with the reported λmax values of these opsins (535, 360, 500, and 370 nm for JSR1, cOpn5m, bovine Rh, and LamPP, respectively) (25, 30, 31), supporting the validity of the methods. Using the same methods, the peak wavelengths of RhRh1, RhRh2, RhRh3, and RhOpn3 were estimated to be 517, 493, 457, and 489 nm, respectively (Fig. 4). The differences in peak wavelengths between RhRh1, RhRh2, and RhRh3 correlate well with their phylogenetic classification (*i*.*e*., LWS, MWS, and SWS/UVS groups). These results, together with the absorption spectra of RhOpn5 and RhPeropsin, indicate that *R*. *hybisae* opsins all show maximum sensitivities in the visible light region, with peak wavelengths ranging from 457 to 517 nm.

### Two subgroups of crustacean Opn5 showing UV and visible light sensitivities

**Figure 5:**
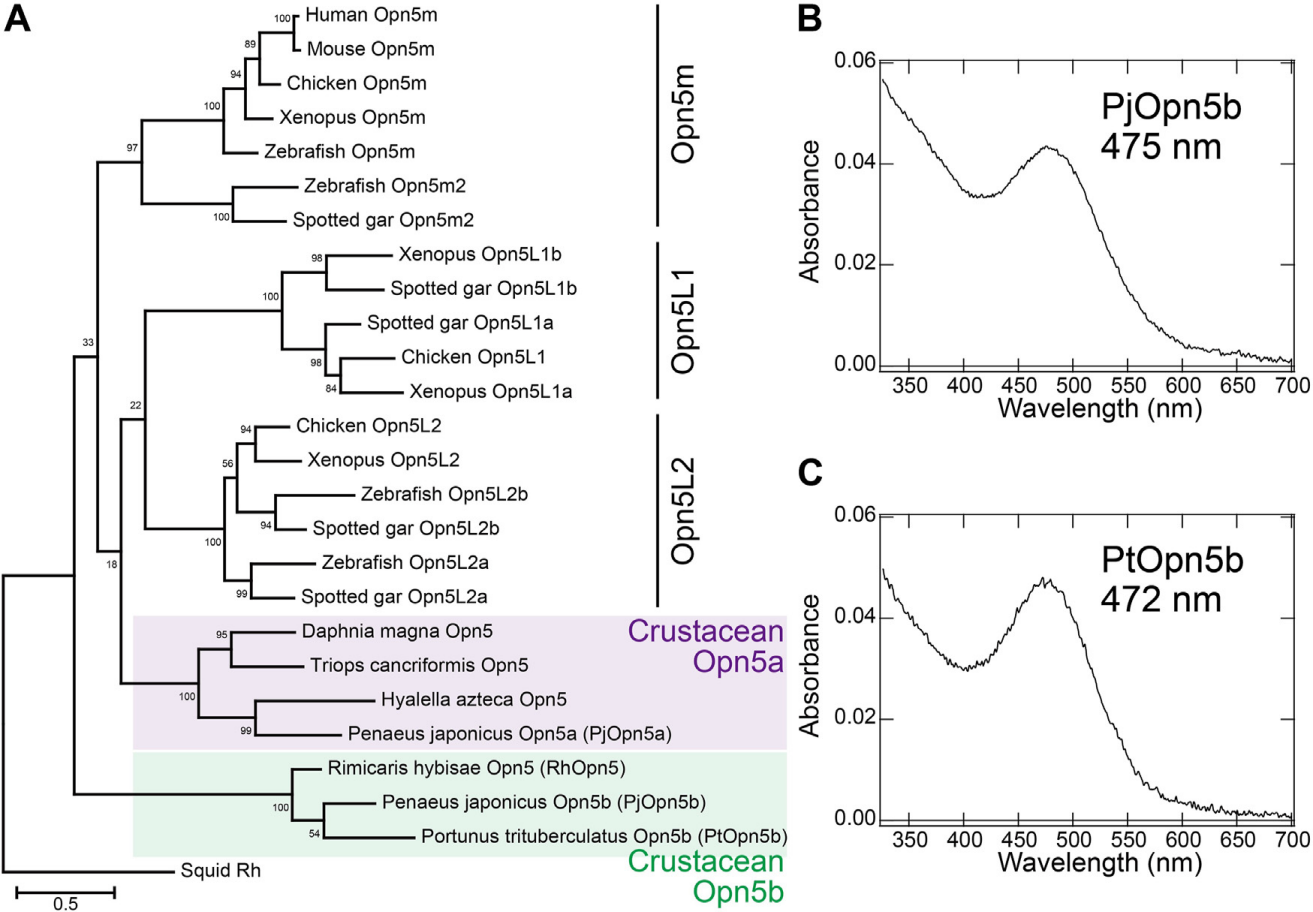
**Visible light sensitivities of crustacean Opn5b**. *A*, phylogenetic relationship of vertebrate Opn5 (Opn5m, Opn5L1, and Opn5L2) and crustacean Opn5. *B* and *C*, absorption spectra of PjOpn5b (*B*) and PtOpn5b (*C*) reconstituted with 11-*cis*-retinal. PjOpn5b, Penaeus japonicus Opn5b; PtOpn5b, Portunus trituberculatus Opn5b.

Since RhOpn5 surprisingly shows visible light sensitivity, we investigated whether this sensitivity is a unique feature specific to the deep-sea habitat of *R*. *hybisae* in contrast with other crustaceans. A search for Opn5 genes in crustaceans showed that crustacean Opn5 opsins are classified into two subgroups: crustacean Opn5a and Opn5b (Fig. 5*A*). To characterize the spectral sensitivity of the crustacean Opn5a subgroup, to which RhOpn5 does not belong, we purified the recombinant proteins of Opn5a from *Penaeus japonicus*, a shallow-water shrimp (PjOpn5a). The absorption spectrum of PjOpn5a reconstituted with 11-*cis*-retinal showed an absorption around 350 nm (Fig. S11*A*). HPLC analysis confirmed that this pigment predominantly bound 11-*cis*-retinal (Fig. S11*D*). These results imply that PjOpn5a forms a UV-sensitive pigment that binds 11*-cis*-retinal as a resting state. UV light (360 ± 10 nm) irradiation induced a spectral red shift to form a photoproduct with a λmax of around 500 nm, accompanied by the conversion from 11-*cis*- to all-*trans*-retinal (Fig. S11). Subsequent yellow light (>500 nm) irradiation induced a spectral blue shift to the UV region, accompanied by the conversion from all-*trans*- to 11*-cis*-retinal (Fig. S11). The resulting spectrum was identical in shape to that observed before irradiation (Fig. S11*A*). The difference spectra calculated before and after UV and yellow light irradiation were mirror images of each other (Fig. S11*B*). These results imply that the resting state and photoproduct are interconvertible by light irradiation, which is a characteristic of bistable opsins, as observed in vertebrate Opn5m and Opn5L2 (25, 26, 27). Therefore, we concluded that PjOpn5a is a UV-sensitive bistable opsin like vertebrate Opn5m and Opn5L2.

To characterize the spectral sensitivity of the crustacean Opn5b subgroup, which includes RhOpn5, we purified recombinant Opn5b proteins from *P*. *japonicus* and *Portunus trituberculatus* (PjOpn5b and PtOpn5b, respectively), both of which are shallow water crustaceans. The absorption spectra of PjOpn5b and PtOpn5b reconstituted with 11-*cis*-retinal showed absorption around 470 nm, similar to RhOpn5 (Fig. 5, *B* and *C* and S12). HPLC analysis confirmed that these pigments predominantly bound 11-*cis*-retinal, with a small amount of 9-*cis* retinal (Fig. S12). Upon yellow light (>480 nm) irradiation, the pigments formed photoproducts around 460 to 470 nm, accompanied by the conversion from 11-*cis*- to all-*trans*-retinal (Fig. S12). These results imply that PjOpn5b and PtOpn5b, like RhOpn5, form a blue light–sensitive resting state. Thus, visible light sensitivity is thought to be a common feature in the crustacean Opn5b subgroup, regardless of habitat (whether shallow or deep-sea environments). In summary, both typical UV-sensitive and atypical visible light–sensitive Opn5 opsins (crustacean Opn5a and Opn5b, respectively) are present in crustacean species.

### Molecular mechanism of the visible light sensitivity of crustacean Opn5b

**Figure 6:**
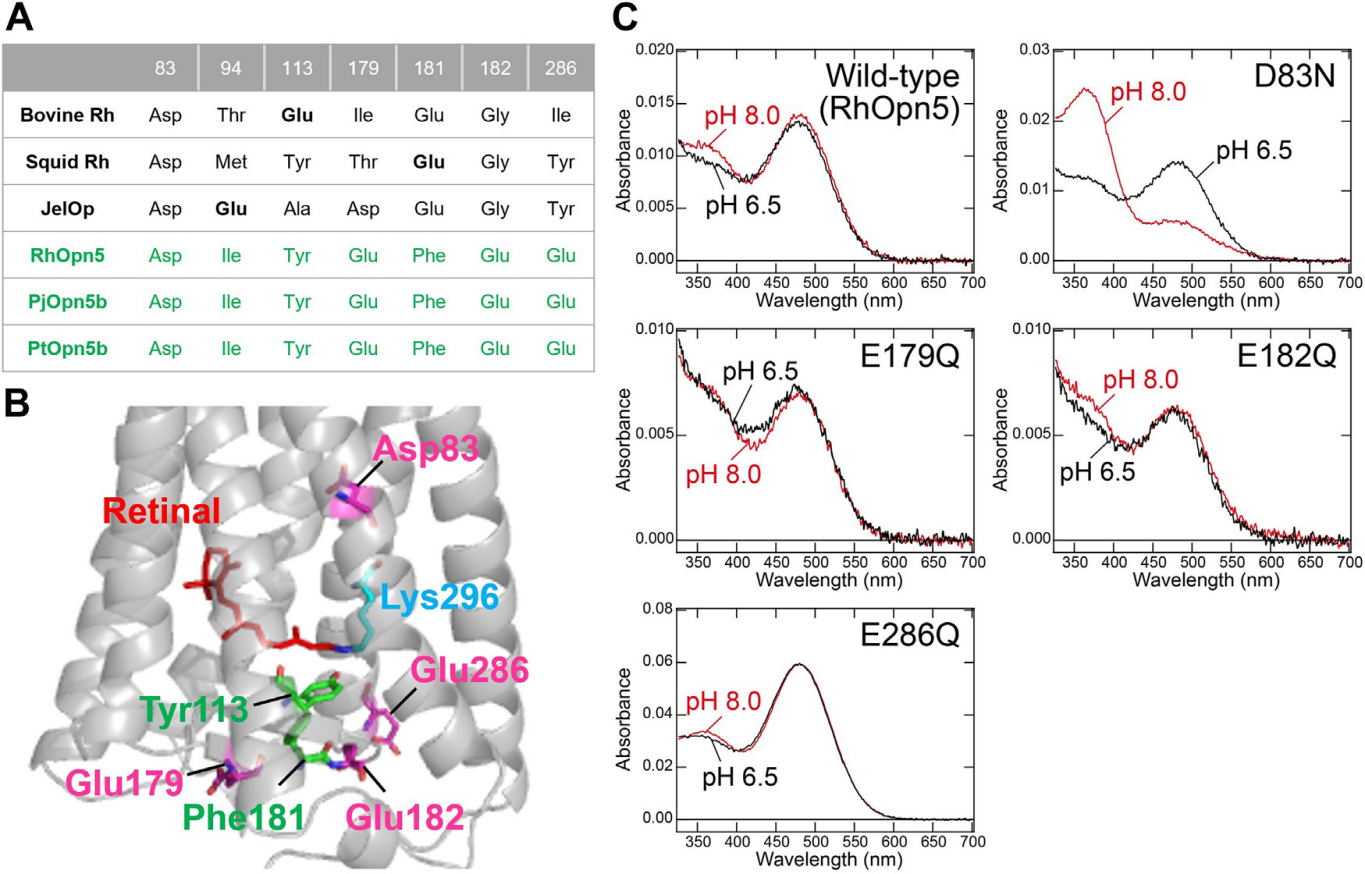
**Counterion residue of crustacean Opn5b**. *A*, comparison of amino acid residues of bovine Rh, squid Rh, JelOp, RhOpn5, PjOpn5b, and PtOpn5b. *B*, model structure of RhOpn5 constructed by AlphaFold. The mutated residues (Asp83, Glu179, Glu182, and Glu286), Lys296, and 11-*cis*-retinal are highlighted in *magenta*, *blue*, and *red*, respectively. The residues at positions 113 and 181, which are typical counterion sites in opsins, are highlighted in *green*. *C*, absorption spectra of wildtype RhOpn5 and the D83N, E179Q, E182Q, and E286Q mutants of RhOpn5. The absorption spectra were measured at pH 8.0 and 6.5. Opn5, Rimicaris hybisae Opn5; PjOpn5b, Penaeus japonicus Opn5b; PtOpn5b, Portunus trituberculatus Opn5b.

To clarify the molecular mechanism of the unique visible light sensitivity of the crustacean Opn5b subgroup, we sought to identify the counterion residue. It is well known that the Glu residue at position 181 in the second extracellular loop (ECL2) functions as a counterion in many types of opsins, such as Gq-coupled opsin, Go-coupled opsin, peropsin, and retinochrome (29, 32, 33). In fact, Glu181 is conserved in RhRh1, RhRh2, RhRh3, RhOpn3, and RhPeropsin, strongly suggesting that these *R*. *hybisae* opsins utilize Glu181 as their counterion (Fig. S2). On the other hand, certain opsins have counterions at positions other than 181. Vertebrate visual opsins (rhodopsin and cone pigment) and *Leptochiton asellus* xenopsin possess the counterion residue at position 113 (Glu113) in the third transmembrane helix (34, 35, 36, 37), whereas JelOp has the counterion residue at position 94 (Glu94) in the second transmembrane helix (38). A comparison of amino acid sequences of crustacean Opn5b opsins (*i*.*e*., RhOpn5, PjOpn5b, and PtOpn5b) reveals that they lack a negatively charged residue (*i*.*e*., Glu and Asp) at positions 94, 113, and 181 (Fig. 6, *A* and *B*), suggesting that this subgroup utilizes a counterion at a different position. To identify the counterion position, we examined amino acid residues located within 15 Å of the retinal chromophore in the crystal structure of bovine Rh in RhOpn5, PjOpn5b, and PtOpn5b. This analysis identified four conserved carboxylate residues, at positions 83, 179, 182, and 286 (*i*.*e*., Asp83, Glu179, Glu182, and Glu286) (Fig. 6, *A* and *B*). To determine which carboxylate residue functions as a counterion, we prepared the recombinant proteins of single mutants of RhOpn5 (*i*.*e*., D83N, E179Q, E182Q, and E286Q), where each residue was substituted with a polar uncharged one. The absorption spectrum of the wildtype RhOpn5 was nearly unaffected by pH changes between 6.5 and 8.0 (Fig. 6*C*). Similarly, the E179Q, E182Q, and E286Q mutants did not show pH-dependent spectral changes in the absorption spectra within this pH range (Fig. 6*C*). In contrast, the D83N mutant showed a significant pH-dependent change, with a small peak in the visible region at pH 8.0 (Fig. 6*C*). This implies a considerable decrease in the Schiff base p*K*a in the D83N mutant, as observed in counterion mutants of other opsins such as the E113Q mutant of bovine Rh and the E181Q mutant of JSR1 (29, 32, 33, 34). Thus, these data indicate that Asp83 functions as a counterion in RhOpn5. Given that Asp83 is conserved in crustacean Opn5b, it is highly likely that all crustacean Opn5b opsins utilize Asp83 as a counterion to achieve visible light sensitivity.

## Discussion

As crustaceans occupy a wide range of habitats and light environments (*e*.*g*., shallow and deep sea, burrowing habitats, and caves), their photoreceptor systems are thought to be diversified and adapted to their environmental photic conditions (39). It has been reported that three groups of Gq-coupled visual opsins (*i*.*e*., LWS, MWS, SWS/UVS) and five groups of nonvisual opsins (*i*.*e*., Rh7, arthropsin, Opn3, Opn5, and peropsin) are present in decapod crustaceans (18, 39). Although the spectral sensitivity of arthropsin remains unclear, Rh7 is recognized as a UV-sensitive opsin (40). This suggests an evolutionary scenario in which *R*. *hybisae* has lost UV-sensitive opsin genes, such as Rh7 and UV-sensitive Opn5 (crustacean Opn5a), because of the near absence of UV light around hydrothermal vents, but has preferentially maintained visible light–sensitive opsin genes (*i*.*e*., three Gq-coupled visual opsins, Opn3, peropsin, and atypical visible light–sensitive Opn5 [crustacean Opn5b]) to detect dim visible light. The convergence of visible light–sensitive opsins in *R*. *hybisae* offers a distinct advantage for efficiently detecting dim surrounding light (Fig. S13). However, further genomic analysis of *R*. *hybisae* is needed to corroborate this hypothesis, as the apparent absence of UV-sensitive opsin transcripts may be due to RNA instability or low expression levels. We confirmed that RhRh1, RhRh3, and RhOpn3 are expressed in the dorsal eye (Fig. S3). Considering that RhRh1 and RhRh3 are categorized as visual opsins and activate Gq-type G proteins (Figs. 2 and S7), which is a central cascade of the crustacean visual signal transduction system, *R*. *hybisae* presumably utilizes these opsins for visual perception in the dorsal eye. While black-body radiation consists of visible light with a relatively long wavelength (>500 nm) (11), the bioluminescence from marine bacteria, which serves as a marker for shrimp bait, generally consists of blue light (approximately 440–500 nm) (8) (Fig. S13). Given that RhRh1 and RhRh3 show red- and blue-shifted spectral sensitivities (λmax = 517 and 457 nm, respectively) (Fig. 4), it is likely that *R*. *hybisae* recognizes the lethal area near the vents and the feeding area containing marine bacteria by detecting red- and blue-shifted visible light with RhRh1 and RhRh3, respectively. On the other hand, we speculate that RhOpn3 is responsible for nonvisual light perception *via* activation of Gi and/or Gi signaling in the dorsal eye. Furthermore, RhRh2 was expressed in the brain but not the dorsal eye, suggesting that RhRh2 is also involved in nonvisual light perception in the brain. It has been reported that crustacean opsins are responsible for various types of nonvisual functions such as daily vertical migrations, reproductive processes, molting behavior, and gonadal maturation (41, 42). The hydrothermal mussel, like shallow-water mussels, has demonstrated the ability to respond to the environmental light cycle (43). This may suggest the presence of an unknown daily or seasonal light cycle around hydrothermal vents, although there is no experimental evidence to support this. Therefore, we propose that *R*. *hybisae* utilizes RhOpn3 and RhRh2 to sense environmental light conditions and thereby perform nonvisual functions. The significant expression of RhOpn5 and RhPeropsin was not detected in the dorsal eye, brain, or antenna, which implies that the expression levels of these opsins are lower than those of other opsins in adults. Further transcriptomic analysis of *R*. *hybisae* at different developmental stages, such as larvae and postlarvae, may provide insights into the expression patterns of RhOpn5 and RhPeropsin and their physiological roles. Recent transcriptomic analysis of other deep-sea shrimp species, such as *Alvinocaris longirostris* and *Shinkaicaris leurokolos*, found that these species possess Gq-coupled visual opsins categorized into the LWS and MWS groups; however, the presence of other nonvisual opsins, with the exception of Opn3 in *A*. *longirostris*, remains unclear (44, 45). Further investigation of nonvisual opsins in other hydrothermal vent crustacean species will be necessary to reveal their nonvisual functions at the molecular level.

Our molecular analysis revealed that some crustacean species, including *R*. *hybisae*, possess the unique Opn5, which utilizes Asp83 in the second transmembrane helix as a counterion. Considering that many vertebrate Opn5 opsins and PjOpn5a exhibit UV sensitivity despite the phylogenetic distance between vertebrates and invertebrates, it is likely that an ancestral Opn5 also exhibited UV sensitivity. Thus, it can be proposed that crustacean Opn5b, including RhOpn5, achieved visible light sensitivity uniquely through the acquisition of an Asp83 counterion from an ancestral UV-sensitive Opn5. In opsins with either Glu113 or Glu181 counterions, Ser186 in the ECL2 forms a hydrogen-bonding network that involves the counterion residue and plays central roles in the stabilization of the protonated Schiff base (29, 46). However, in crustacean Opn5b, the residue corresponding to Ser186 is deleted and Glu181 is replaced with a Phe residue, indicating that the local structure around the protonated Schiff base and ECL2 differs significantly from that of typical opsins with Glu113 and Glu181 counterions. Due to the structural differences in the ECL2, crustacean Opn5b probably employs a strategy to utilize Asp83, which is well conserved in many types of opsins, as a counterion. Based on the crystal structure of bovine Rh and JSR1, Asp83 is located on the opposite side (*i*.*e*., intracellular side) relative to the protonated Schiff base, compared with Glu113 and Glu181 (*i*.*e*., extracellular side) (Fig. 6*B*) (46, 47). We speculate that the protonated Schiff base linkage of crustacean Opn5b is oriented to the intracellular side to form the salt bridge with the counterion Asp83. Recently, two Opn5 proteins from the terrestrial slug (*Limax valentianus*) have been reported to show visible light sensitivity with significant UV sensitivity (48). Since *Limax* Opn5 proteins have Glu181 and are phylogenetically distinct from crustacean Opn5b, we speculate that these Opn5 proteins have acquired visible light sensitivity by different molecular mechanisms. Opn5b is present in crustacean species from diverse habitats with very different light conditions (*e*.*g*., shallow and deep sea environments) and commonly absorbs blue light. In the deep sea, blue light is primarily derived from bioluminescence, and in shallower waters, it is predominant because of the scattering and absorption of sunlight by water molecules. This suggests a shared importance of blue light reception mediated by Opn5b across both shallow and deep sea crustacean species. Further molecular and physiological analyses of Opn5b will be essential to clarify its physiological roles.

In conclusion, we characterized visible light–sensitive opsins expressed in the dorsal eye and brain of the hydrothermal vent shrimp *R*. *hybisae*. This shrimp appears to have adapted by preferentially maintaining a repertoire of visible light–sensitive opsins, including the unique visible light–sensitive Opn5, to efficiently detect dim visible light in the deep-sea environment. In addition to our molecular analysis of opsins, further physiological studies of *R*. *hybisae* and other hydrothermal vent crustacean species will provide a deeper understanding of their adaptive strategies for utilizing dim light in the deep-sea habitat.

## Experimental procedures

### Animal sampling and histological analysis

Using the Human Occupied Vehicle *Shinkai6500*, we collected *R*. *hybisae* from two hydrothermal fields in the MCSC in the Caribbean Sea: Beebe (at a water depth of 4967 m) and Von Damm (at a water depth of 2295 m). Collections were performed during the YK13-05 cruise of the research vessel *Yokosuka* operated by Japan Agency for Marine-Earth Science and Technology (JAMSTEC) in June 2013. Animals collected from the Von Damm field were still alive after the onboard recovery and were actively swimming in a collecting chamber. Living animals were immediately fixed with RNAlater (Merck). Samples were dissected in RNAlater and stored at −80 °C.

Sample preparation and microscopic observation of the dorsal eye were performed according to previous studies with some modifications (49, 50). For prefixation, dorsal eyes were immersed in 0.1 M PBS (pH 7.4) containing 2% glutaraldehyde and 2% paraformaldehyde for 16 to 18 h. Postfixation was performed in 2% osmium tetroxide for 1.5 h. After washing with PBS, the specimens were dehydrated in a graded ethanol series and embedded in low-viscosity resin (Spurr resin; Polysciences). Then, 400-nm ultrathin sections were prepared using an ultramicrotome (EM-UC 7; Leica) and stained with toluidine blue. The specimens were then observed under an inverted microscope (IX71; Olympus). Eighty-nanometer ultrathin sections were also prepared using the ultramicrotome and stained with uranyl acetate and lead citrate. These specimens were then observed under a transmission electron microscope (H-7650; Hitachi).

### RNA extraction, sequencing, and identification of opsin genes

Total RNA was extracted from four tissues (dorsal organ, brain, first antenna, and second antenna) of five individuals using the RNeasy Mini Kit (Qiagen). The quality and concentration of total RNA were analyzed by gel electrophoresis using a NanoDrop Spectrophotometer (Agilent) and Q-bit (Thermo Fisher). Only samples that met the necessary quality standard were outsourced to Macrogen, Japan, for library construction using the TruSeq RNA Sample Prep Kit v2 (Illumina) and sequencing by HiSeq 2500 (Illumina). Raw short-read sequences are available in the National Center for Biotechnology Information Sequence Read Archive under accession number SRP339896.

The *R*. *hybisae* transcriptome was screened to identify opsin genes. First, sequences containing seven transmembrane domains (PFAM: 7tm_1, 7tm_2, 7tm_3) were extracted, and opsins were identified with hmmsearch (e value <1e^-5^) using a custom-built opsin hidden Markov model. The presence of the residue Lys296, which is a conserved chromophore-binding site in opsins, was used to validate the identified sequences.

### Transcriptome assembly and annotation

The quality of 12 fastq files was tested by FASTQC (http://www.bioinformatics.babraham.ac.uk/projects/fastqc), and adapters and low-quality reads (threshold of quality control = 20) were trimmed using Trimmomatic-v-0.38 (51). The trimmed reads were assembled *de novo* using Trinity-v2.8.4 (52). Assembled transcript isoforms with high similarity (≥95%) were removed with CD-HIT-EST-v4.6 (53, 54). Transcript abundance was estimated using Bowtie2-v2.3.4.3 (55) and RSEM-v1.3.1 (56) by mapping reads back to the transcript assembly. To reduce data complexity, the transcripts were filtered with TransDecoder-v5.5.0 (http://transdecoder.github.io) using the following criteria: (i) open reading frames larger than 70 amino acids, (ii) sequences with HMMER hits against the Pfam database, and (iii) sequences with BLASTP hits against the Swiss-Prot database. Expression filtering was applied using expression levels ≥2 transcripts per million in at least one sample.

### Differential expression analysis

Tag count comparison and baySeq packages were used to normalize count data and identify differentially expressed transcripts between each sample type (57). Transcripts with a false discovery rate <0.05 were defined as differentially expressed transcripts.

### Expression plasmids for opsins and phylogenetic analysis

The complementary DNAs (cDNAs) of *R*. *hybisae* opsins were optimized for human-codon usage and fused to a C-terminal sequence encoding the epitope recognized by the anti–bovine Rh monoclonal antibody Rho1D4 epitope sequence ETSQVAPA (Supplementary data). The fusion product was inserted into the mammalian expression vector pMT4 (58). The cDNA of bovine Rh (K00506) was inserted into the mammalian expression vector pUSRα. The cDNAs of other opsins (JSR-1: AB251846; JelOp: AB435549; LamPP: AB116380; cOpn5m: AB368182; *P*. *japonicus* Opn5a: XM_043000672; *P*. *japonicus* Opn5b: XM_043033134; and *P*. *trituberculatus* Opn5b: XM_045258436) were tagged with the epitope sequence of the anti–bovine Rh monoclonal antibody Rho1D4 at the C terminus and inserted into the mammalian expression vector pCAGGS or pMT4 (59). The opsin cDNAs containing mutations were constructed using the In-Fusion Cloning Kit (Takara Bio) or the SLiCE method (60). To improve the expression level of opsin proteins, we truncated 170 amino acid residues from the N terminus of the RhRh1 protein and 134, 178, 221, 185, and 170 residues from the C termini of RhRh3, RhOpn5, *P*. *japonicus* Opn5a, *P*. *japonicus* Opn5b, and *P*. *trituberculatus* Opn5b proteins, respectively.

The phylogenetic tree was inferred by the maximum-likelihood method of MEGA X software (version 10.2.2) (61) using the Jones–Taylor–Thornton matrix–based model (62). The numbers at each node are bootstrap probabilities estimated by 1000 replications. Accession numbers of the sequence data in Figures 1 and 5 are as follows: bovine Rh, K00506; human Rh, NP_000530; human red, Z68193; human blue, M13299; salmon VA opsin, NP_001117098; LamPP, AB116380; human encephalopsin, AF140242; zebrafish Opn3, EF043381; medaka TMT1A, JX293354; mosquito Opn3, BAN05625; amphioxus Rh, AB050606; scallop SCOP2, AB006455; human Opn4, AF147788; zebrafish Opn4m2, AY078161; amphioxus melanopsin, AB205400; human RGR, AH005747; squid retinochrome, X57143; human peropsin, AF012270; amphioxus peropsin, AB050610; jumping spider peropsin, AB525082; chicken Opn5L2, AB368183; human Opn5m, AY377391; hydrozoan JelOp, AB332435; box JelOp, AB435549; *Acropora millepora* Acropsin 1, MK829324; *A*. *millepora* Acropsin 6, MK829329; *Owenia fusiformis* xenopsin, MF133511; *L*. *asellus* xenopsin, MF133514; scallop SCOP1, XP_021357501; octopus Rh, X07797, squid Rh, P31356; *Branchinella kugenumaensis* SWS, AB293436; *Procambarus clarkii* SWS, T304797; *Platyeriocheir formosa* SWS, KT693122; *Eriocheir japonica* SWS, KT693119; *Homarus americanus* SWS, XP_042206726; *P*. *formosa* MWS, KT693121; *Eriocheir japonica* MWS, KT693117; *Uca vomeris* MWS, GQ228847; *Squilla empusa* LWS, GQ221753; *P*. *clarkii* LWS, KT304796; *Neomysis americana* LWS, DQ852592; *Cambarellus schufeldtii* LWS, AF003544, human beta-2 adrenergic receptor, AAA88015; mouse Opn5m, AY318865; cOpn5m AB368182; *Xenopus* Opn5m, XM_002935990; zebrafish Opn5m, AY493740; zebrafish Opn5m2, XM_005157939; spotted gar Opn5m2, XM_015337843; chicken Opn5L1, AB368181; *Xenopus* Opn5L1a, XM_002934130; *Xenopus* Opn5L1b, NM_001079378; spotted gar Opn5L1a, XM_015339535; spotted gar Opn5L1b, XM_015364771; *Xenopus* Opn5L2, XM_004914948; zebrafish Opn5L2a, XM_001341956; spotted gar Opn5L2a, XM_015343997; zebrafish Opn5L2b, NM_001316945; spotted gar Opn5L2b, XM_015351606; *Daphnia magna* Opn5, KZS13848; *Hyalella azteca* Opn5, XP_018024074; *P*. *japonicus* Opn5a, XM_043000672; *P*. *japonicus* Opn5b, XM_043033134; and *P*. *trituberculatus* Opn5b, XM_045258436.

### Ca^2+^ and cAMP level measurement in cultured cells

Intracellular Ca^2+^ and cAMP levels in cultured cells were measured using aequorin- and Glosensor-based bioluminescence assays according to previous studies, with some modifications (19, 63). HEK293T cells were seeded in 96-well plates at 22,000 cells per well in Dulbecco’s modified Eagle's medium/Nutrient Mixture F-12 (Gibco, Thermo Fisher Scientific), supplemented with 10% fetal bovine serum, 0.0625% (w/v) penicillin, and 0.01% (w/v) streptomycin. After incubation for 24 h, the cells were transfected with 100 ng of opsin plasmid and 100 ng of aequorin or Glosensor 22F plasmid per well by the polyethyleneimine transfection method (19). After incubation for 5 to 6 h, 11-*cis*-retinal (for all opsins except RhPeropsin) or all-*trans*-retinal (for RhPeropsin) was added to the medium (final concentration: 5 μM). PTX, where used, was added to the retinal media at 100 ng ml^-1^. After overnight incubation, the medium was replaced with the following: for the aequorin assay, with equilibration medium, Leibovitz's L-15 Medium (Gibco, Thermo Fisher Scientific) containing coelenterazine h; and for the Glosensor assay, with CO_2_-independent medium (Gibco, Thermo Fisher Scientific) containing 10% fetal bovine serum and GloSensor cAMP reagent stock solution. After 2 h of incubation in the dark, luminescence was measured using microplate luminometers (Veritas, Turner Biosystems; and GloMax Discover Microplate Reader, Promega). The cells were irradiated with visible light covering wavelengths from 420 to 700 nm through a Y-44 cutoff filter (HOYA) from a white LED source (SLA-10013; OptoSigma) for which the light intensity was adjusted to 3.0 mW cm^-2^. For the measurement of action spectra using the aequorin-based bioluminescence assay, rhodopsin-expressing cells were prepared as described previously. For the measurement of action spectra using the Glosensor-based bioluminescence assay, HEK293T cells were seeded in a 35-mm dish at 600,000 cells per well. After incubation for 24 h, the cells were transfected with 2 μg of opsin plasmid and 2 μg of Glosensor 22F plasmid per well by the polyethylenimine transfection method (19). After incubation for 5 to 6 h, 11-*cis*-retinal was added to the medium (final concentration: 5 μM). After overnight incubation and medium exchange with the equilibration medium, the cells were irradiated with different colors of light generated by letting white LED light pass through band-pass filters (420 ± 10 nm, 450 ± 10 nm, 480 ± 10 nm, 520 ± 10 nm, 550 ± 10 nm, 570 ± 10 nm, 600 ± 12 nm, 640 ± 10 nm, 660 ± 10 nm; Asahi Spectra Co, Ltd) or with UV LED (WindFire) light that was passed through a band-pass filter (360 ± 10 nm; Asahi Spectra Co, Ltd). The quantum flux of each LED light was adjusted to 1.26 × 10^14^ photons cm^-2^ s^-1^.

### NanoBiT G protein dissociation assay in cultured cells

NanoBiT G protein dissociation assay in cultured cells was performed according to previous studies with some modifications (64, 65). The coding sequences of Lg-BiT-inserted human Gqα, Lg-BiT-inserted human Gi1α, human Gβ1, Sm-BiT-fused human Gγ2 (C68S), and RIC8A were constructed and inserted into the pMT vector as described in previous studies (23, 64, 65). HEK293T cells were seeded in 96-well plates as in the aequorin- and GloSensor-based bioluminescence assays. After incubation for 24 h, the cells were transfected with 50 ng of opsin plasmid, 5 ng Lg-BiT-inserted Gqα or Giα (Gqα-LgBiT or Giα-LgBiT) plasmid, 25 ng Gβ1 plasmid, 25 ng Sm-BiT-fused Gγ2 plasmid, and 5 ng of RIC8A plasmid (only for the Gq activation assay) per well by the polyethyleneimine transfection method. After incubation for 5 to 6 h, 11*-cis*-retinal (for all opsins except RhPeropsin) or all-*trans*-retinal (for RhPeropsin) was added to the medium (final concentration: 5 μM). After overnight incubation, the medium was replaced with Leibovitz's L-15 Medium (Gibco, Thermo Fisher Scientific) containing coelenterazine h. After 2 h of incubation in the dark, luminescence was measured using a microplate luminometer (GloMax Navigator Microplate Luminometer; Promega). The cells were irradiated with visible light through a Y-44 cutoff filter (HOYA) from a white LED source (SLA-10013; OptoSigma) for which the light intensity was adjusted to 3.0 mW cm^-2^.

### Expression and purification of opsin recombinant proteins

HEK293T cells were cultured in Dulbecco’s modified Eagle's medium/Nutrient Mixture F-12 (Gibco, Thermo Fisher Scientific), supplemented with 10% fetal bovine serum, 0.0625% (w/v) penicillin, and 0.01% (w/v) streptomycin. The expression plasmids were transiently transfected using the calcium phosphate method (66, 67). After 1 day of incubation, 11-*cis*- or all-*trans*-retinal (final concentration: 5 μM) was added to the transfected cells to reconstitute the photoactive pigments. After another day of incubation, the cells were collected by centrifugation (6500*g* for 10 min at 4 °C) and were resuspended in buffer A (50 mM Hepes [pH 7.0] and 140 mM NaCl). The cells were solubilized in buffer A containing 1% *n*-dodecyl-β-d-maltoside (DOJINDO) and adsorbed to a Rho1D4 affinity column to purify the pigments. After the column was washed with buffer A containing 0.02% *n*-dodecyl-β-d-maltoside, the pigment was eluted by adding synthetic peptide containing the epitope sequence.

### Western blotting

Western blotting analysis was performed according to previous studies with some modifications (68, 69). Extracts from opsin-transfected HEK293 cells were subjected to SDS-PAGE (12.5%), transferred to a polyvinylidene difluoride membrane, and probed with the monoclonal antibody Rho1D4. Immunoreactive proteins were detected using the ECL Prime Western Blotting System (Cytiva) and visualized with a luminescent image analyzer (LuminoGraph; ATTO).

### Spectroscopic measurement and HPLC analysis

The absorption spectra of the samples were recorded with a UV–visible spectrophotometer (Shimadzu UV-2600). Samples were kept at 0 °C using a cell holder equipped with a circulation system that used temperature-controlled water. The samples were irradiated with either yellow light (>500 or >480 nm, passed through a Y-52 or Y-50 cutoff filter [HOYA], respectively) from a white LED source (SLA-10013; OptoSigma) or with UV light (360 ± 10 nm, passed through a band-pass filter [Asahi Spectra Co Ltd]) from a handheld UV flashlight (WindFire). Chromophore configurations of the samples were analyzed by HPLC as previously described (68). The molar compositions of the retinal isomers were calculated from the areas of the peaks in the HPLC patterns monitored at 360 nm and from extinction coefficients of each isomer as previously described (68, 70).

## Data availability

All data are available in the main text or the supporting information.

## Supporting information

This article contains supporting information.

## Conflict of interest

The authors declare that they have no conflicts of interest with the contents of this article.
