# Supplementary material for: A repertoire of visible light–sensitive opsins in the deep-sea hydrothermal vent shrimp Rimicaris hybisae
Source: J Biol Chem. 2025 May 26;301(7):110291. doi: 10.1016/j.jbc.2025.110291 (PMC12221356; doi:10.1016/j.jbc.2025.110291)
Supplement: Supporting Information [file mmc1.docx]

Supporting information for

A repertoire of visible light-sensitive opsins in the deep-sea hydrothermal vent shrimp *Rimicaris hybisae*

Yuya Nagata^1^, Norio Miyamoto^2*^, Keita Sato^3^, Yosuke Nishimura^4^, Yuki Tanioka^5^, Yuji Yamanaka^5^, Susumu Yoshizawa^6^, Kuto Takahashi^1^, Kohei Obayashi^7^, Hisao Tsukamoto^7^, Ken Takai^2^, Hideyo Ohuchi^3^, Takahiro Yamashita^8^, Yuki Sudo^3^, Keiichi Kojima^3*^

^1^ Graduate School of Medicine, Dentistry and Pharmaceutical Sciences, Okayama University, Okayama 700‑8530, Japan.

^2^ Institute for Extra‑Cutting‑Edge Science and Technology Avant‑Garde Research (X‑Star), Japan Agency for Marine-Earth Science and Technology (JAMSTEC), Kanagawa 237-0061, Japan.

^3^ Faculty of Medicine, Dentistry and Pharmaceutical Sciences, Okayama University, Okayama 700-8530, 700-8558, Japan.

^4^ Research Center for Bioscience and Nanoscience (CeBN), Research Institute for Marine Resources Utilization, Japan Agency for Marine-Earth Science and Technology (JAMSTEC), Kanagawa 237-0061, Japan.

^5^ School of Pharmaceutical Sciences, Okayama University, Okayama 700-8530, Japan.

^6^ Atmosphere and Ocean Research Institute, The University of Tokyo, Chiba 277-8564, Japan.

^7^ Department of Biology, Graduate School of Science, Kobe University, Kobe, Japan.

^8^ Department of Biophysics, Graduate School of Science, Kyoto University, Kyoto 606-8502, Japan.


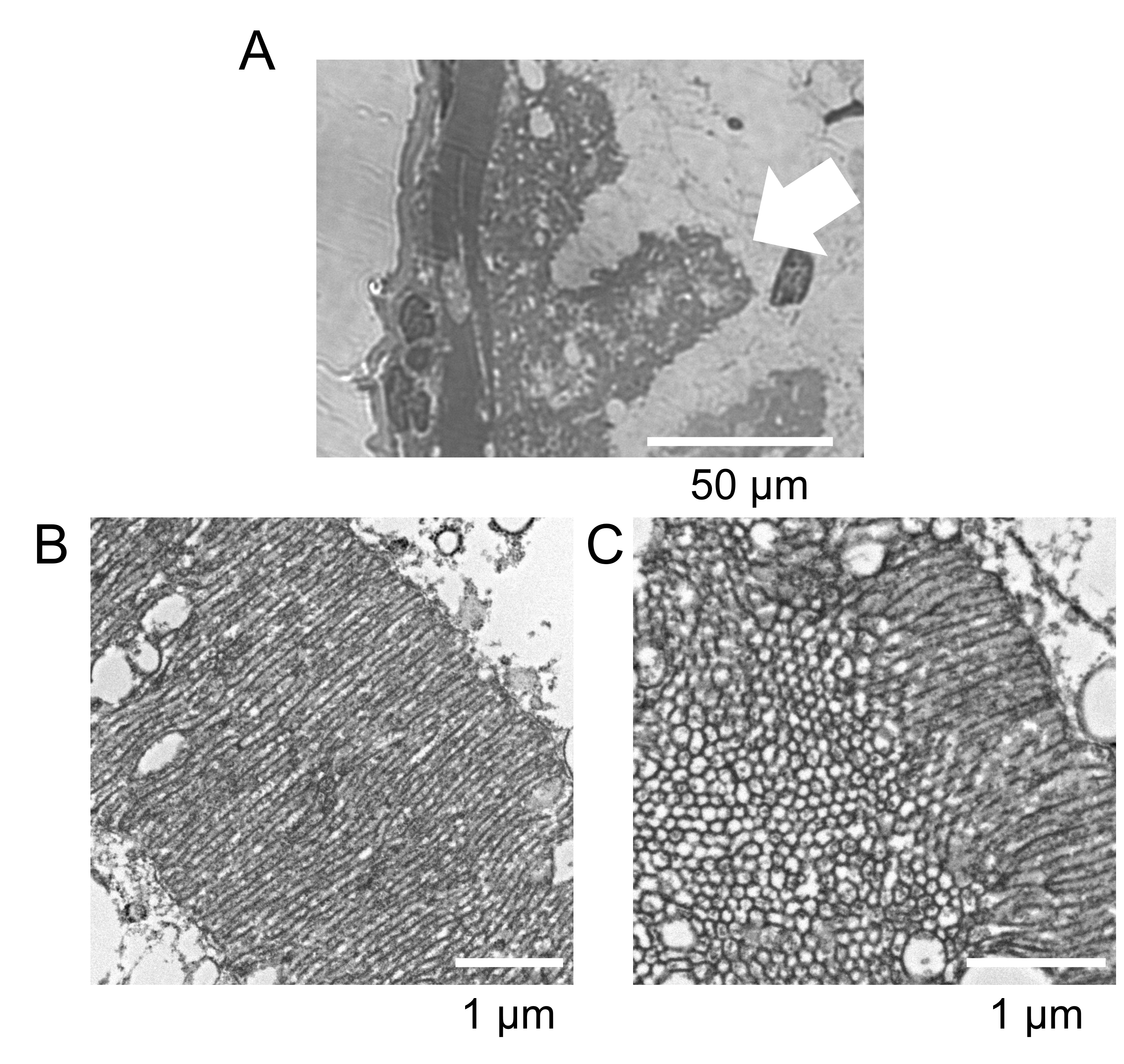
 **Supplementary Figures**

**Figure S1 Morphology of the dorsal eye of *R. hybisae*.** (A) Light micrographs of a plastic section of the dorsal eye stained with toluidine blue. The white arrow indicates an enlarged rhabdomeral segment. (B, C) The internal abutted array and cross-section of microvilli of rhabdomeric photoreceptors.


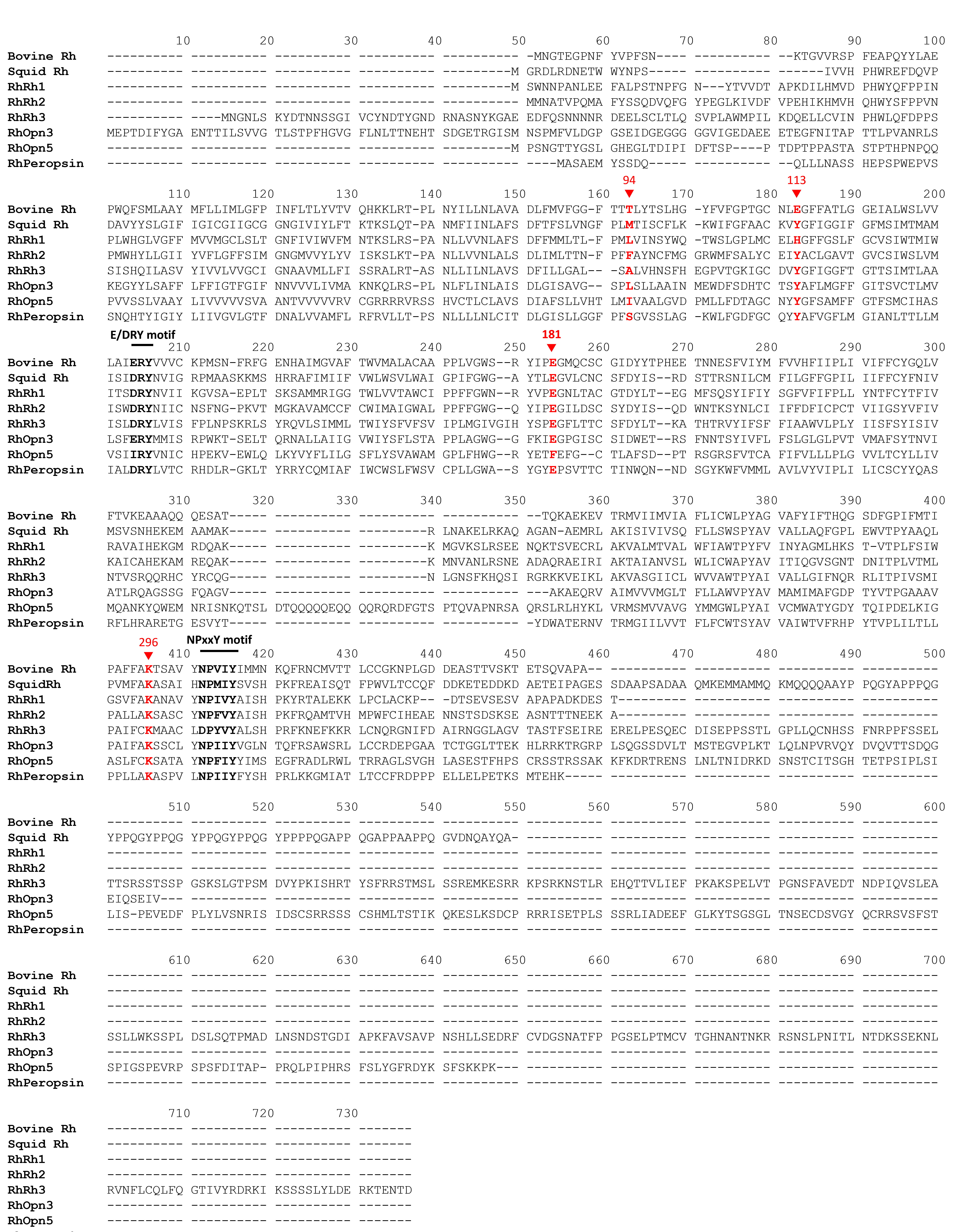
**Figure S2 Amino acid sequence alignment of *R. hybisae* opsins.** Multiple alignments of opsin sequences from *R. hybisae*, bovine rhodopsin (bovine Rh), and squid rhodopsin (squid Rh). Amino acid residues at 94, 113, 181, and 296 are colored red. Functionally important motifs (E/DRY and NPxxY motifs) are also shown. The bovine rhodopsin numbering system was used in this study.


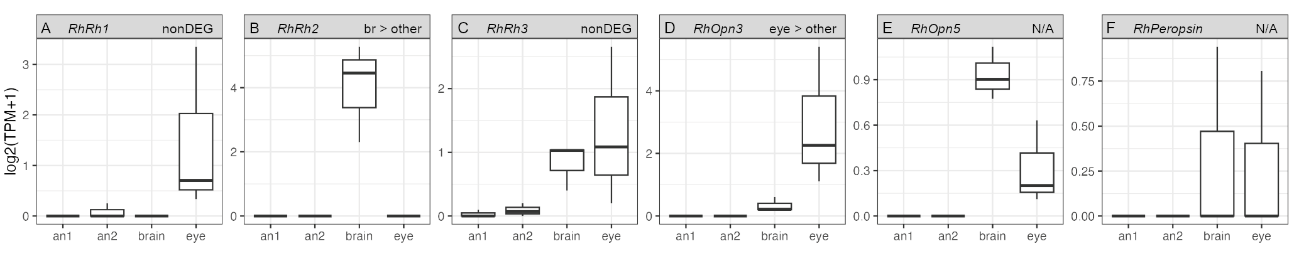
**Figure S3 Expression levels of *R. hybisae* opsins.** Box plots of opsin expression across samples (log2(TPM+1)). The text at the upper right of each panel indicates the result of TCC-baySeq analysis: nonDEG, not differentially expressed; br > other, differentially expressed in the brain; eye > other, differentially expressed in the eye; N/A, not applicable (meaning the expression levels of all samples are under the threshold). RhRh2 was expressed in the brain. RhOpn3, RhRh1, and RhRh3 were expressed in the dorsal eye.


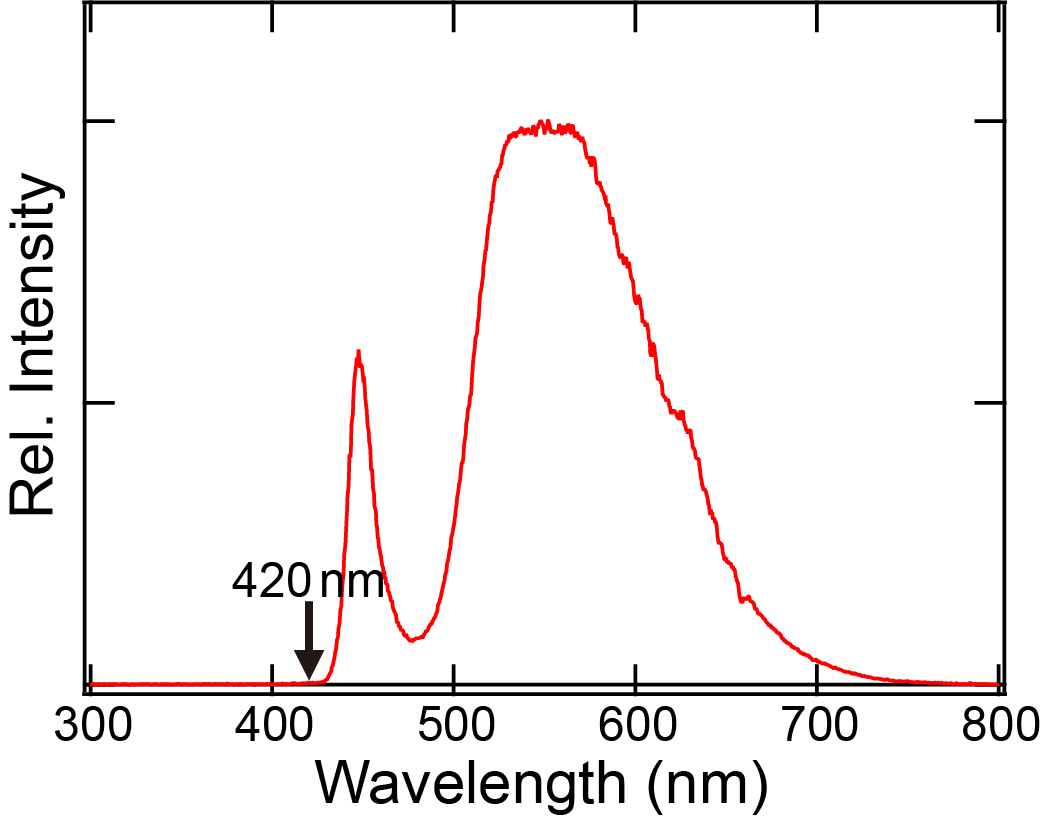
**Figure S4 Irradiance spectrum of visible light used to activate opsin-expressing cells**. Visible light was generated by passing white LED light (SLA-10013, OptoSigma) through a Y-44 cutoff filter (HOYA). The spectrum was measured using a USB2000+ spectrophotometer (OceanOptics).


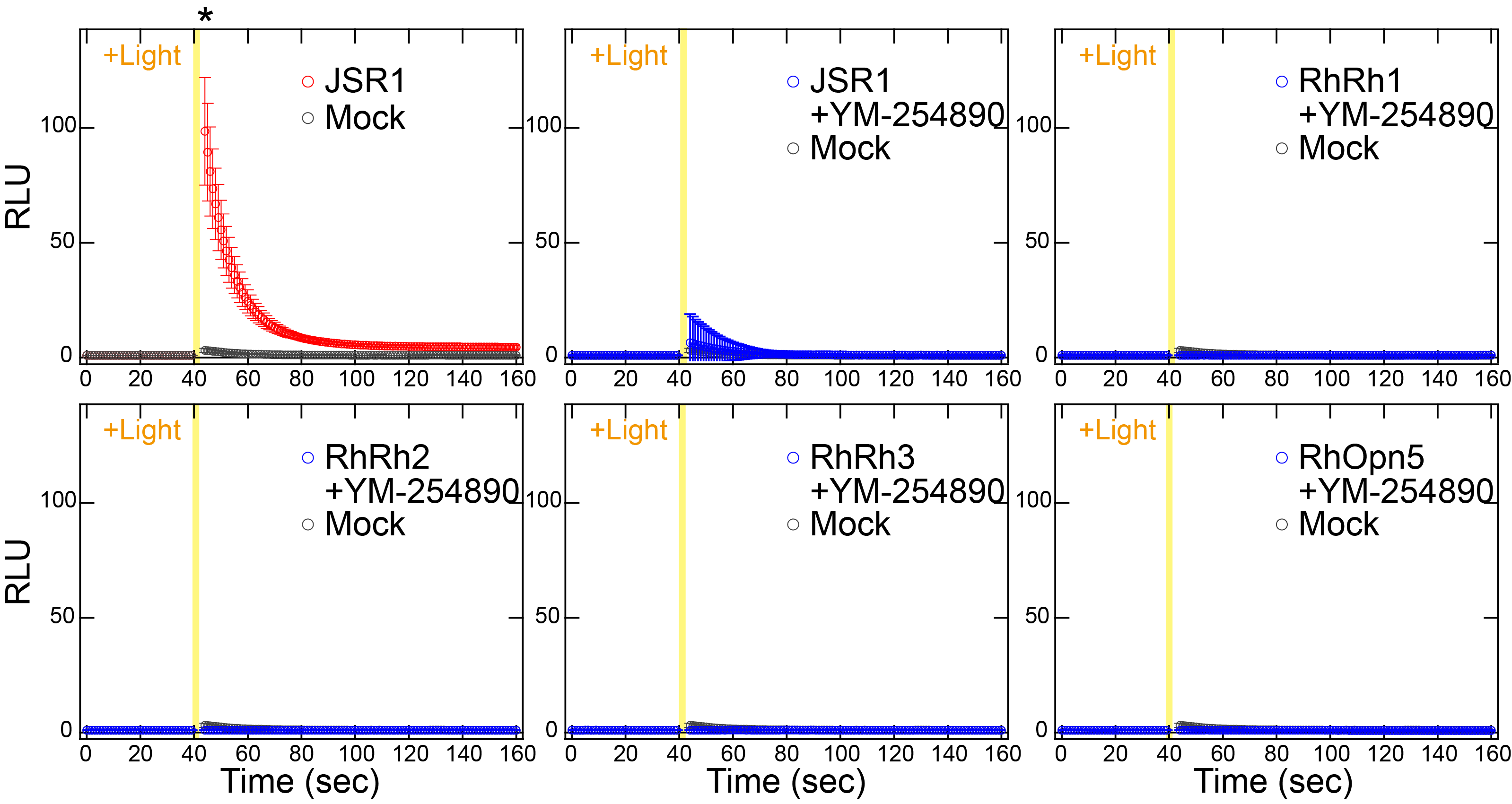
**Figure S5 Light-induced changes in intracellular Ca^2+^ levels for each opsin.** The Ca^2+^ levels in JSR1-, RhRh1-, RhRh2-, RhRh3-, RhOpn5-, and mock-transfected HEK293 cells were measured using the aequorin assay. The cells were irradiated with visible light covering wavelengths from 420 to 700 nm for 3 s, and treated with 1 μM YM-254890 (Gq inhibitor) prior to the irradiation. Yellow vertical lines indicate the timing of visible light irradiation to the cells. Data are presented as means ± SD from more than three independent experiments. * indicates a significant difference in luminescence values after irradiation between opsin- and mock-transfected HEK293 cells (p < 0.05; Dunnett’s test). RLU stands for relative light units.


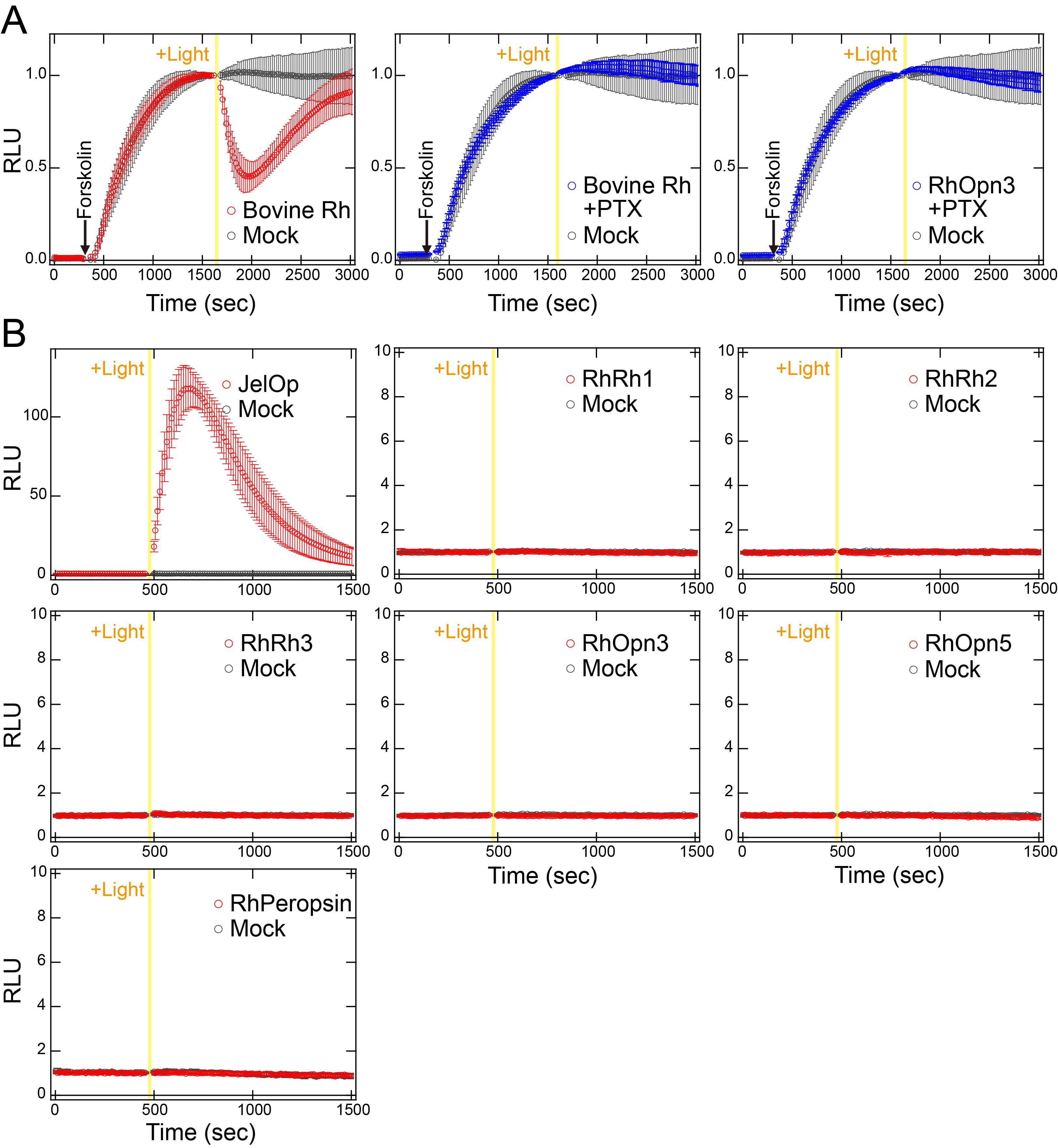
**Figure S6 Light-induced changes in intracellular cAMP levels for each opsin.** (A) The cAMP levels in bovine Rh-, RhOpn3-, and mock-transfected HEK293 cells, treated or untreated with PTX, were measured using the GloSensor cAMP assay. Cells were treated with 2 μM forskolin prior to irradiation with visible light covering wavelengths from 420 to 700 nm for 30 s. (B) The cAMP levels in JelOp-, RhRh1-, RhRh2-, RhRh3-, RhOpn3-, RhOpn5-, RhPeropsin-, and mock-transfected HEK293 cells, treated with PTX, were measured using the GloSensor cAMP assay to evaluate Gs activity. Cells were irradiated with visible light covering wavelengths from 420 to 700 nm for 20 s. Yellow vertical lines in all panels of this figure indicate the timing of visible light irradiation to the cells. Data are presented as the means ± SD from more than three independent experiments. RLU stands for relative light units.


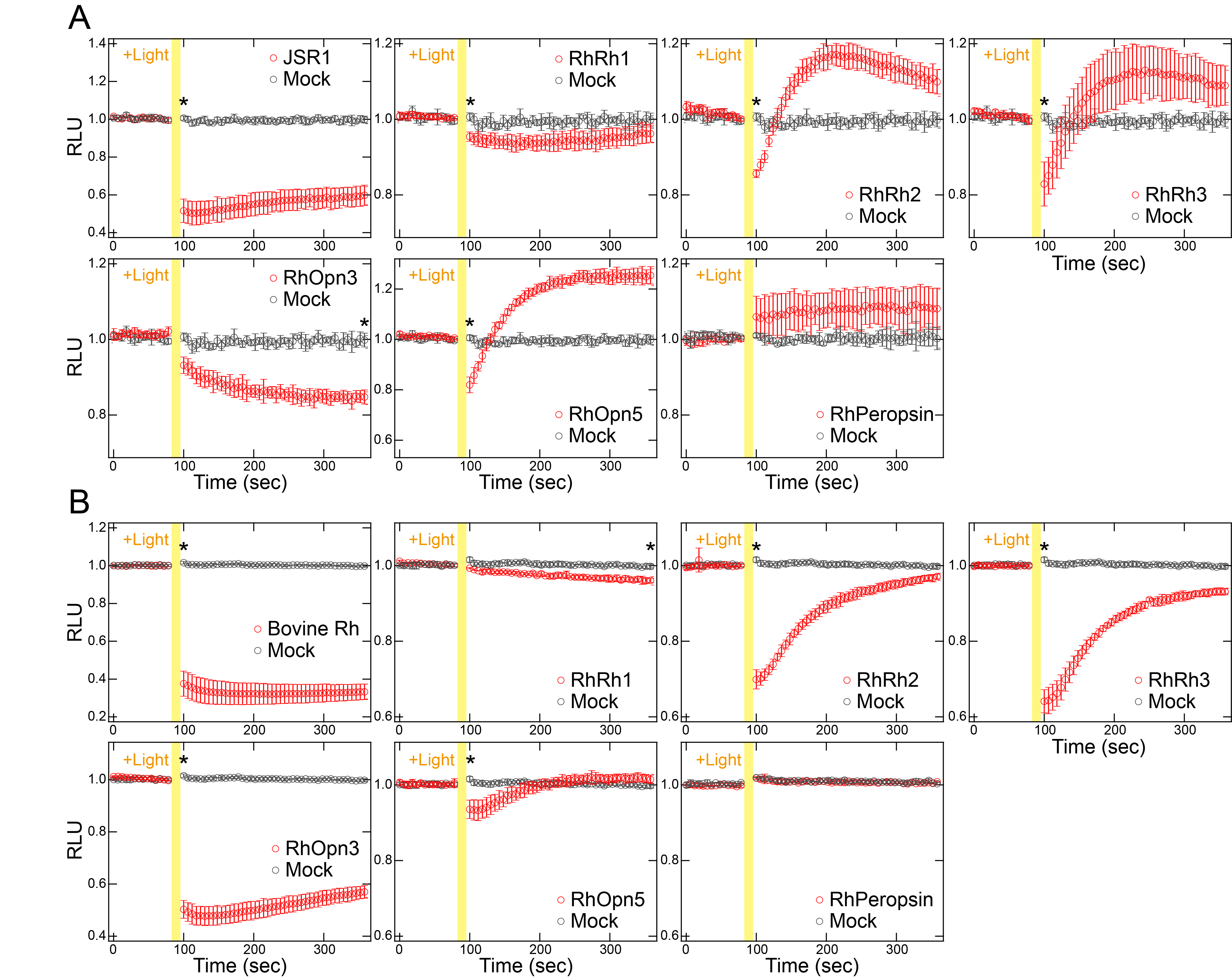
**Figure S7 NanoBiT G protein dissociation assay for each opsin.** Luminescence changes of NanoBiT Gq (A) and Gi (B) proteins in opsin- and mock-transfected HEK293 cells. The cells were irradiated with visible light covering wavelengths from 420 to 700 nm for 20 s. Yellow vertical lines indicate the timing of visible light irradiation to the cells. Data are presented as means ± SD from more than three independent experiments. RLU stands for relative light units. * indicates a significant difference in luminescence values either immediately after or 294 s after irradiation between opsin- and mock-transfected HEK293 cells (p < 0.05; Dunnett’s test).


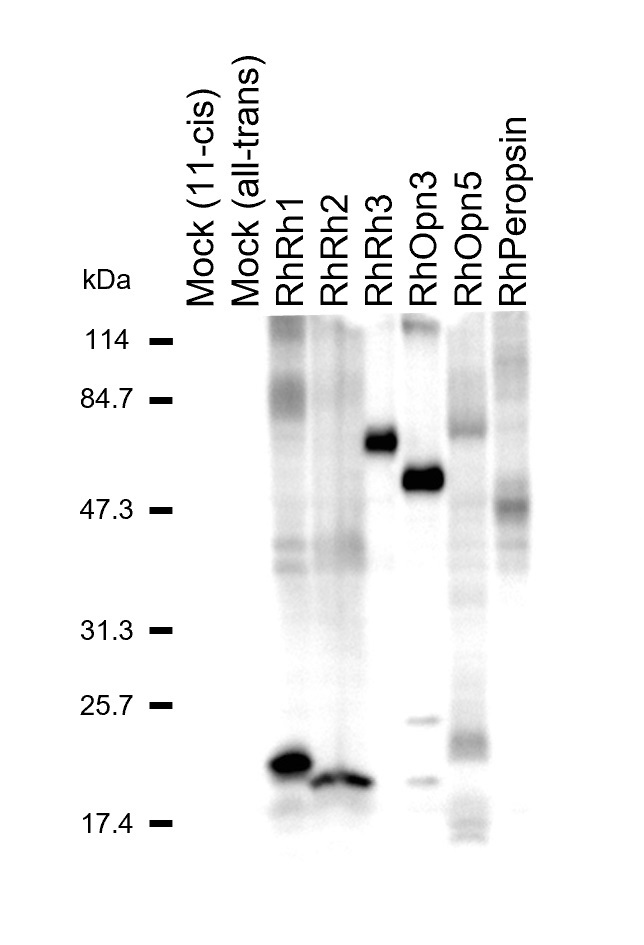
**Figure S8 Western blotting analysis of *R. hybisae* opsins expressed in HEK293 cells.** HEK293 cells transfected with each Rimicaris opsin were subjected to Western blotting using the monoclonal antibody Rho1D4. Mock-transfected cells incubated with 11-cis-retinal and all-trans-retinal were included as negative controls. The calculated molecular weights of the Rimicaris opsins are as follows: RhRh1, 43 kDa; RhRh2, 44 kDa; RhRh3, 63 kDa; RhOpn3, 51 kDa; RhOpn5, 46 kDa; RhPeropsin, 40 kDa. Recombinant opsins showed multiple bands above their calculated molecular weights probably because of their oligomerization and the heterogeneity in posttranslational modification within the cultured cells.


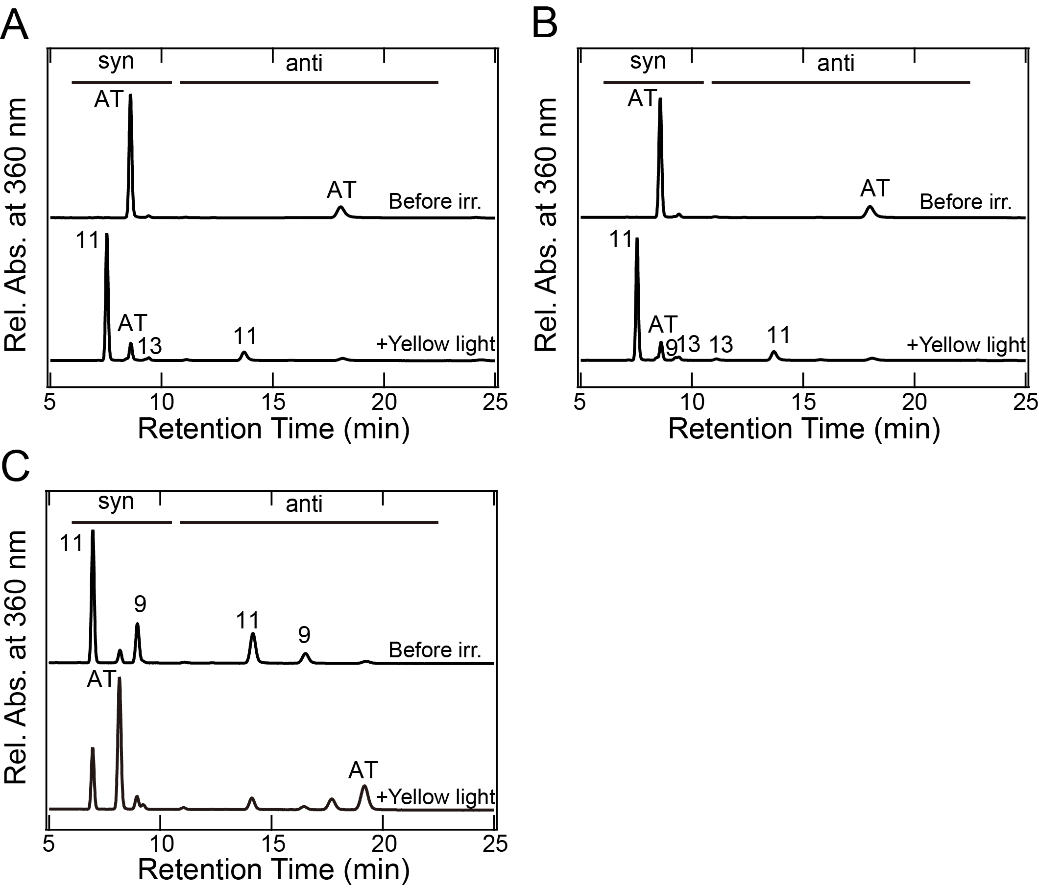
**Figure S9 HPLC analysis of light-induced retinal configuration changes of RhPeropsin and RhOpn5.** (A, B) Retinal configuration changes caused by yellow light (>500 nm) irradiation of RhPeropsin reconstituted with all-trans- (A) and 11-cis-retinal (B). (C) Retinal configuration changes caused by yellow light (>480 nm) irradiation of RhOpn5 reconstituted with 11-cis-retinal. Retinal configurations before and after light irradiation were determined by HPLC after extracting the chromophore as retinal oximes: *syn* and *anti* forms of 11-cis-retinal oximes (11), 13-cis-retinal oximes (13), 9-cis-retinal oximes (9), and all-trans-retinal oximes (AT).


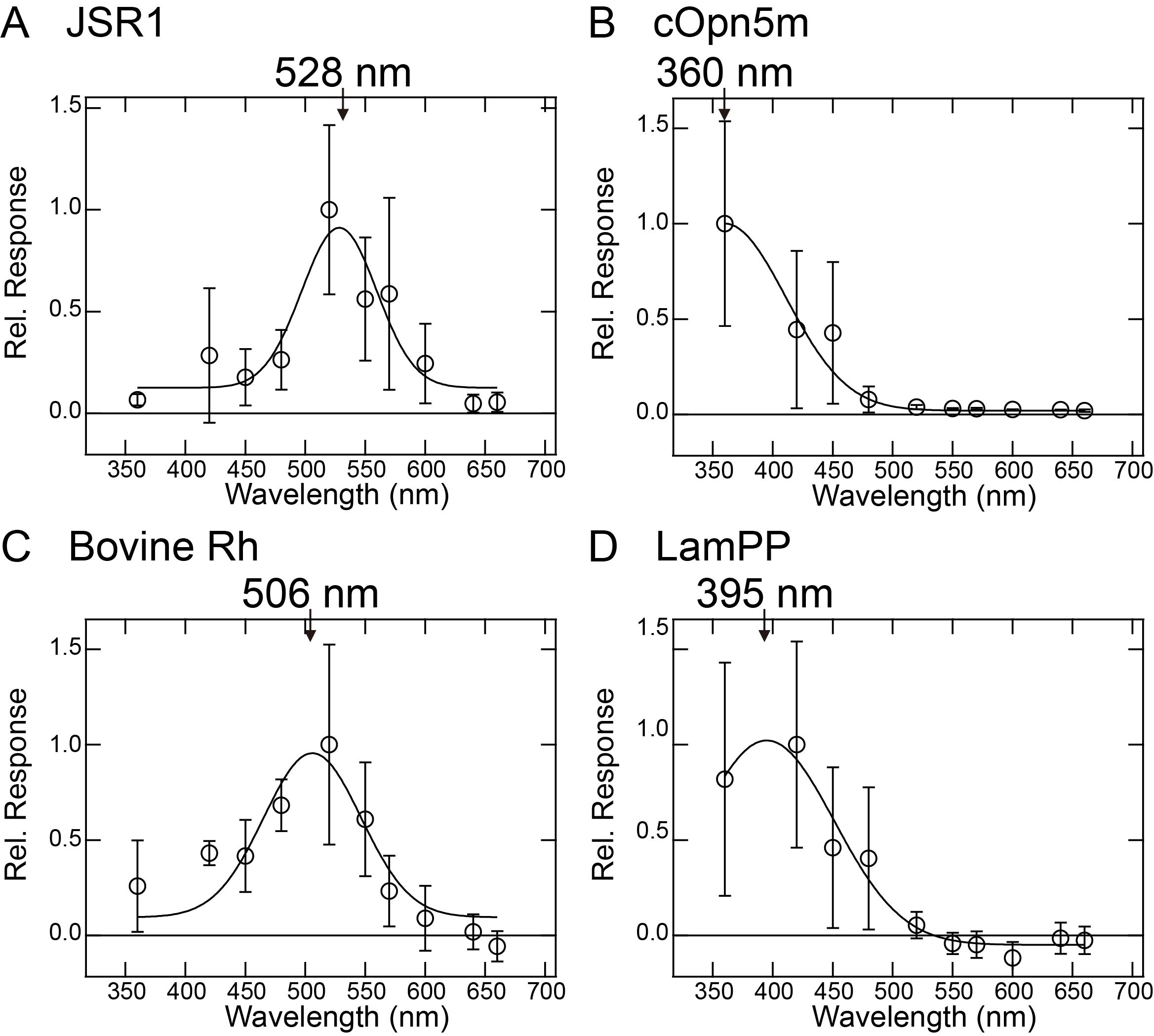
**Figure S10 Spectral sensitivities of opsins.** The relative light-induced responses of JSR1 (A), cOpn5m (B), bovine Rh (C), and LamPP (D). The responses were determined by the amplitudes of light-induced changes in intracellular Ca^2+^ levels in JSR1- and cOpn5m-transfected HEK293 cells, and by the initial slopes of light-induced changes in cAMP levels in bovine Rh- and LamPP-transfected HEK293 cells (please see the Materials and Methods section for details). Data are presented as the means ± SD from more than three independent experiments. The responses were fitted with a Gaussian curve to estimate the peak wavelength (528, 360, 506, and 395 nm for JSR1, cOpn5m, bovine Rh, and LamPP, respectively).


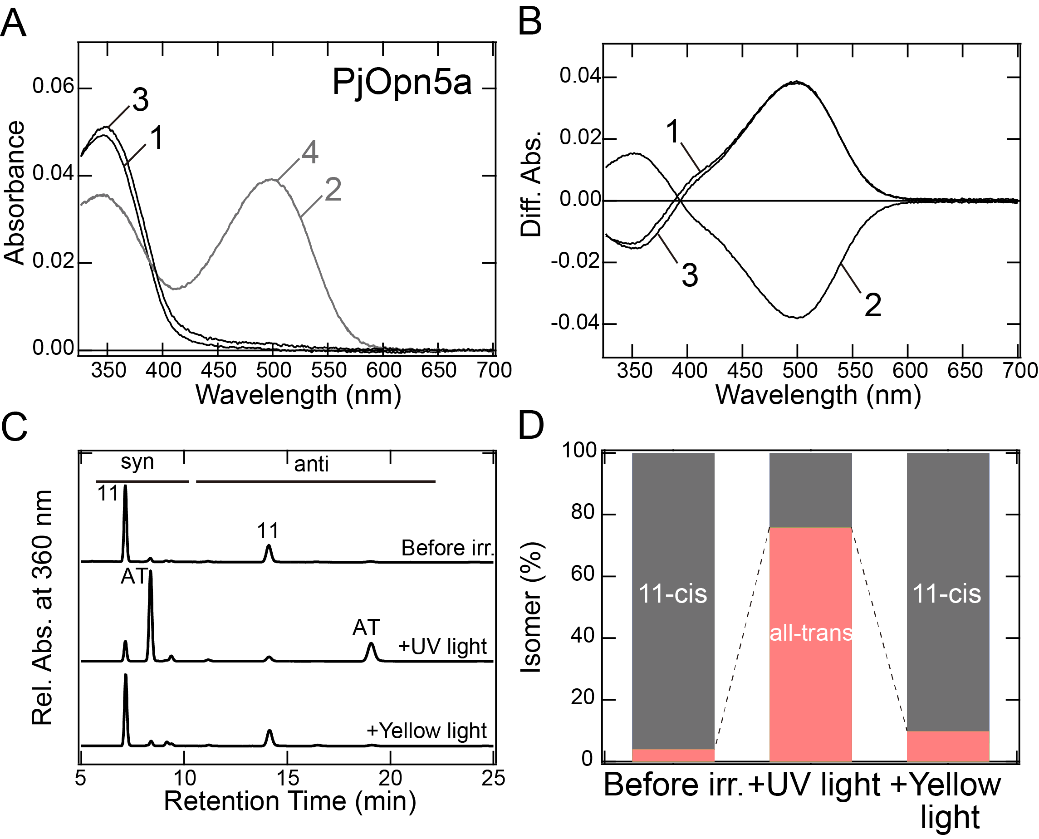
**Figure S11 Photochemical properties of PjOpn5a.** (A) Absorption spectra of PjOpn5a reconstituted with 11-cis-retinal. Absorption spectra of PjOpn5a were measured in the dark (curve 1), after UV light irradiation (360 ± 10 nm) (curve 2), after subsequent yellow light irradiation (>500 nm) (curve 3), and after subsequent UV light (360 ± 10 nm) reirradiation (curve 4). (B) Spectral changes caused by the initial UV light irradiation (curve 1), and subsequent yellow light (curve 2) and subsequent UV reirradiation (curve 3). (C) Retinal configuration changes caused by UV and subsequent yellow light irradiation of PjOpn5a reconstituted with 11-cis-retinal. (D) Isomeric compositions of retinal in the dark and after UV and subsequent yellow light irradiation of PjOpn5a were estimated by HPLC analysis.


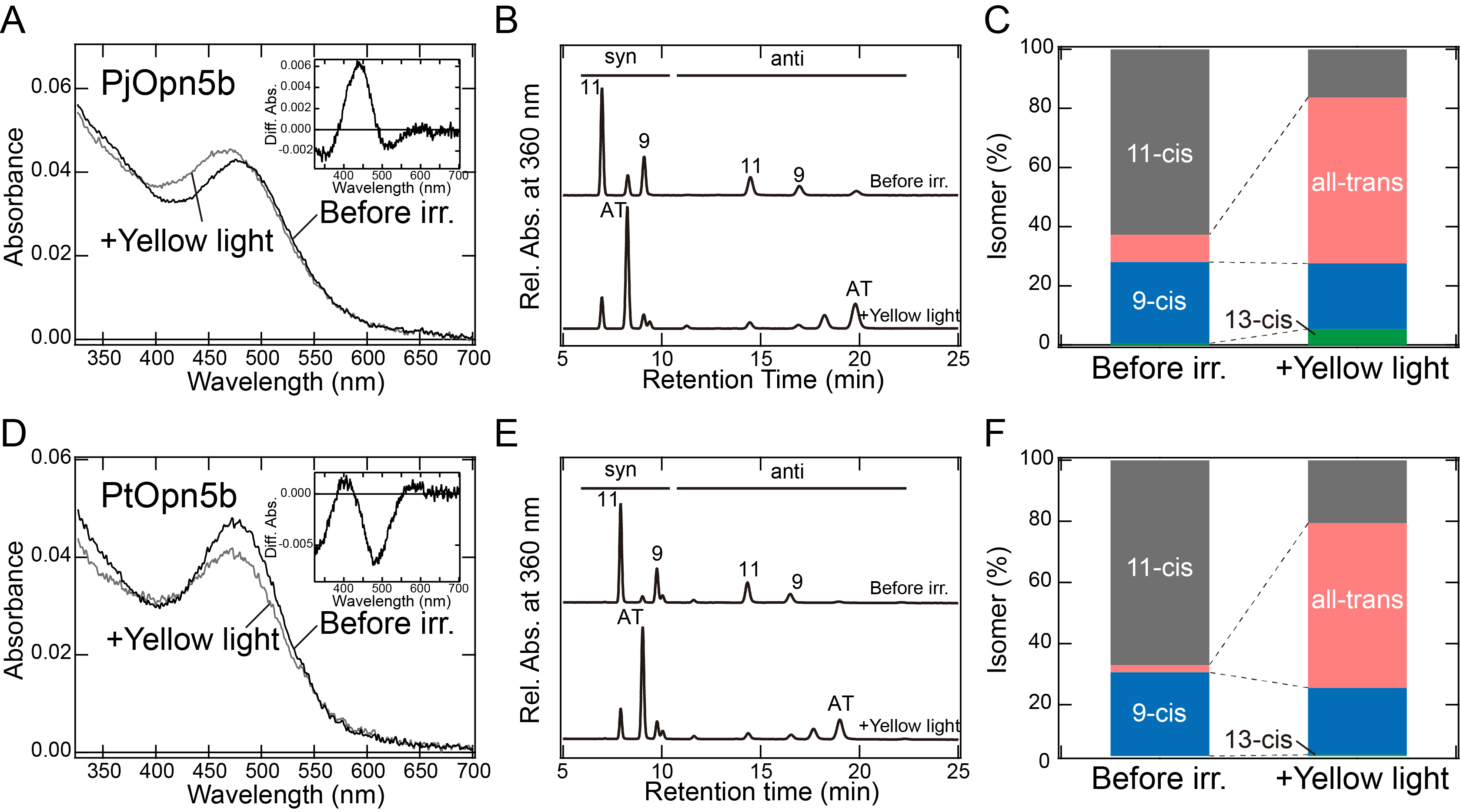
**Figure S12 Photochemical properties of PjOpn5b and PtOpn5b.** (A, D) Absorption spectra of PjOpn5b (A) and PtOpn5b (D) reconstituted with 11-cis-retinal. Black and gray curves indicate the spectra in the dark and after yellow light (>480 nm) irradiation, respectively. (Inset) The curve represents the difference spectra before and after yellow light irradiation. (B, E) Retinal configuration changes caused by yellow light irradiation of PjOpn5b (B) and PtOpn5b (E) reconstituted with 11-cis-retinal. (C, F) Isomeric compositions of retinal in the dark and after yellow light irradiation of PjOpn5b (C) and PtOpn5b (F) were estimated by HPLC analysis.


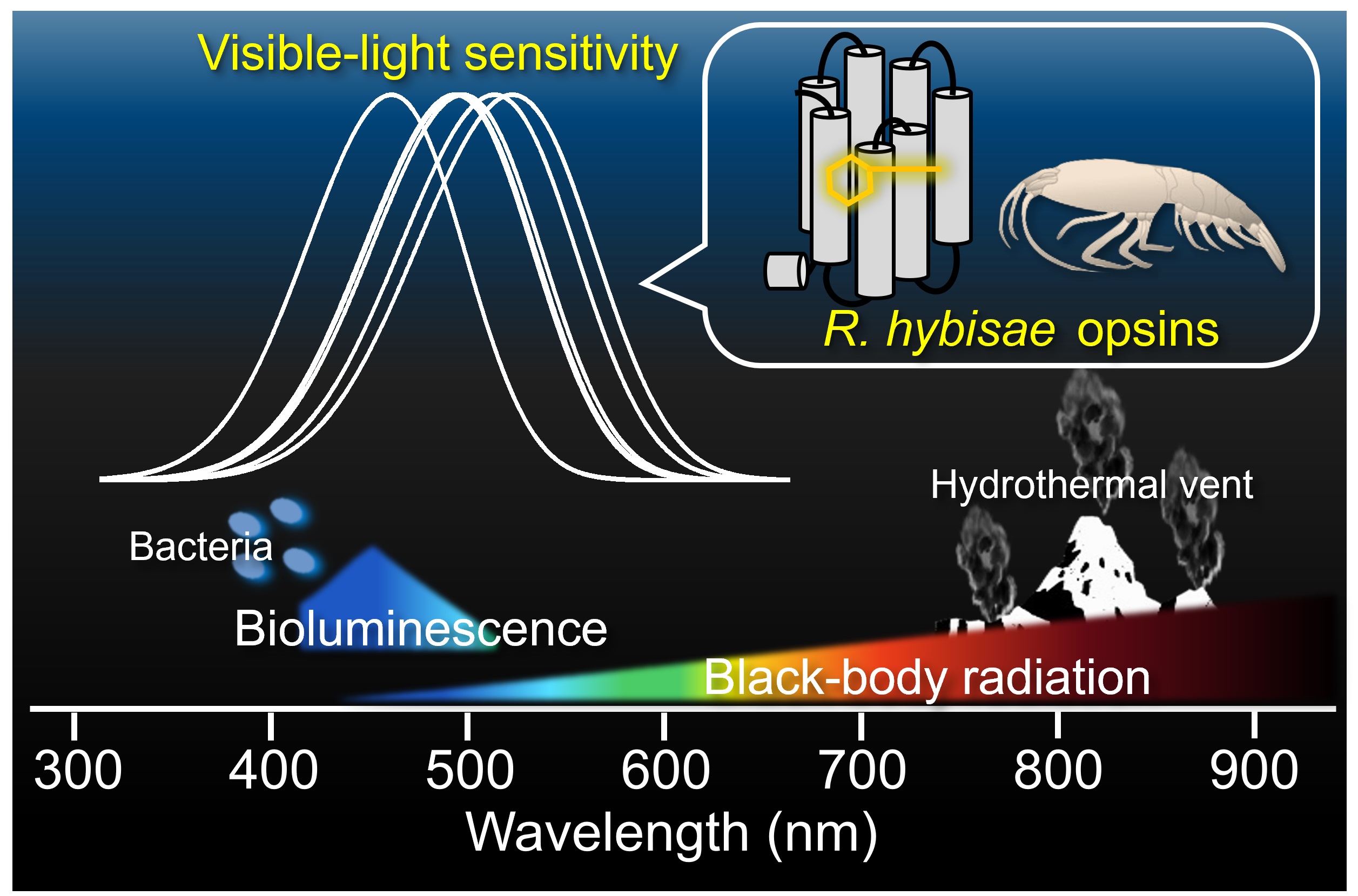
**Figure S13 Schematic relationship between the spectral sensitivities of *R. hybisae* opsins and the emission wavelengths from black-body radiation and bioluminescence.**

**Supplementary data : Amino acid sequences of *R. hybisae* opsins used in this study**

>RhRh1 with the 1D4 tag

MSWNNPANLEEFALPSTNPFGNYTVVDTAPKDILHMVDPHWYQFPPINPLWHGLVGFFMVVMGCLSLTGNFIVIWVFMNTKSLRSPANLLVVNLAFSDFFMMLTLFPMLVINSYWQTWSLGPLMCELHGFFGSLFGCVSIWTMIWITSDRYNVIIKGVSAEPLTSKSAMMRIGGTWLVVTAWCIPPFFGWNRYVPEGNLTACGTDYLTEGMFSQSYIFIYSGFVFIFPLLYNTFCYTFIVRAVAIHEKGMRDQAKKMGVKSLRSEENQKTSVECRLAKVALMTVALWFIAWTPYFVINYAGMLHKSTVTPLFSIWGSVFAKANAVYNPIVYAISHPKYRTALEKKLPCLACKPDTSEVSESVAPAPADKDESTETSQVAPA

Red: the 1D4 tag

>RhRh2 with the 1D4 tag

MMNATVPQMAFYSSQDVQFGYPEGLKIVDFVPEHIKHMVHQHWYSFPPVNPMWHYLLGIIYVFLGFFSIMGNGMVVYLYVISKSLKTPANLLVVNLALSDLIMLTTNFPFFAYNCFMGGRWMFSALYCEIYACLGAVTGVCSIWSLVMISWDRYNIICNSFNGPKVTMGKAVAMCCFCWIMAIGWALPPFFGWGQYIPEGILDSCSYDYISQDWNTKSYNLCIIFFDFICPCTVIIGSYVFIVKAICAHEKAMREQAKKMNVANLRSNEADAQRAEIRIAKTAIANVSLWLICWAPYAVITIQGVSGNTDNITPLVTMLPALLAKSASCYNPFVYAISHPKFRQAMTVHMPWFCIHEAENNSTSDSKSEASNTTTNEEKAETSQVAPA

Red: the 1D4 tag

>RhRh3 with the 1D4 tag

MNGNLSKYDTNNSSGIVCYNDTYGNDRNASNYKGAEEDFQSNNNNRDEELSCLTLQSVPLAWMPILKDQELLCVINPHWLQFDPPSSISHQILASVYIVVLVVGCIGNAAVMLLFISSRALRTASNLLILNLAVSDFILLGALSALVHNSFHEGPVTGKIGCDVYGFIGGFTGTTSIMTLAAISLDRYLVISFPLNPSKRLSYRQVLSIMMLTWIYSFVFSVIPLMGIVGIHYSPEGFLTTCSFDYLTKATHTRVYIFSFFIAAWVLPLYIISFSYISIVNTVSRQQRHCYRCQGNLGNSFKHQSIRGRKKVEIKLAKVASGIICLWVVAWTPYAIVALLGIFNQRRLITPIVSMIPAIFCKMAACLDPYVYALSHPRFKNEFKKRLCNQRGNIFDAIRNGGLAGVTASTFSEIREERELPESQECDISEPPSSTLGPLLQCNHSSFNRPPFSSELTTSRSSTSSPGSKSLGTPSMDVYPKISHRTYSFRRSTMSLSSREMKESRRKPSRKNSTLREHQTTVLIEFPKAKSPELVTPGNSFAVEDTNDPIQVSLEASSLETSQVAPA

Red: the 1D4 tag

>RhOpn3 with the 1D4 tag

MEPTDIFYGAENTTILSVVGTLSTPFHGVGFLNLTTNEHTSDGETRGISMNSPMFVLDGPGSEIDGEGGGGGVIGEDAEEETEGFNITAPTTLPVANRLSKEGYYLSAFFLFFIGTFGIFNNVVVLIVMAKNKQLRSPLNLFLINLAISDLGISAVGSPLSLLAAINMEWDFSDHTCTSYAFLMGFFGITSVCTLMVLSFERYMMISRPWKTSELTQRNALLAIIGVWIYSFLSTAPPLAGWGGFKIEGPGISCSIDWETRSFNNTSYIVFLFSLGLGLPVTVMAFSYTNVIATLRQAGSSGFQAGVAKAEQRVAIMVVVMGLTFLLAWVPYAVMAMIMAFGDPTYVTPGAAAVPAIFAKSSCLYNPIIYVGLNTQFRSAWSRLLCCRDEPGAATCTGGLTTEKHLRRKTRGRPLSQGSSDVLTMSTEGVPLKTLQLNPVRVQYDVQVTTSDQGEIQSEIVETSQVAPA

Red: the 1D4 tag

>RhOpn5 with the 1D4 tag

MPSNGTTYGSLGHEGLTDIPIDFTSPPTDPTPPASTASTPTHPNPQQPVVSSLVAAYLIVVVVVSVAANTVVVVVRVCGRRRRVRSSHVCTLCLAVSDIAFSLLVHTLMIVAALGVDPMLLFDTAGCNYYGFSAMFFGTFSMCIHASVSIIRYVNICHPEKVEWLQLKYVYFLILGSFLYSVAWAMGPLFHWGRYETFEFGCTLAFSDPTRSGRSFVTCAFIFVLLLPLGVVLTCYLLIVMQANKYQWEMNRISNKQTSLDTQQQQQEQQQQRQRDFGTSPTQVAPNRSAQRSLRLHYKLVRMSMVVAVGYMMGWLPYAIVCMWATYGDYTQIPDELKIGASLFCKSATAYNPFIYYIMSEGFRADLRWLTRRAGLSVGHLASESTFHPSCRSSTRSSAKKFKDRTRETSQVAPA

Red: the 1D4 tag

>RhPeropsin with the 1D4 tag

MASAEMYSSDQQLLLNASSHEPSPWEPVSSNQHTYIGIYLIIVGVLGTFDNALVVAMFLRFRVLLTPSNLLLLNLCITDLGISLLGGFPFSGVSSLAGKWLFGDFGCQYYAFVGFLMGIANLTTLLMIALDRYLVTCRHDLRGKLTYRRYCQMIAFIWCWSLFWSVCPLLGWASYGYEPSVTTCTINWQNNDSGYKWFVMMLAVLVYVIPLILICSCYYQASRFLHRARETGESVYTYDWATERNVTRMGIILVVTFLFCWTSYAVVAIWTVFRHPYTVPLILTLLPPLLAKASPVLNPIIYFYSHPRLKKGMIATLTCCFRDPPPELLELPETKSMTEHKETSQVAPA

Red: the 1D4 tag
